# Expansion and functional divergence of terpene synthase genes in angiosperms: a driving force of terpene diversity

**DOI:** 10.1093/hr/uhae272

**Published:** 2024-09-25

**Authors:** Qi Wang, Jie Jiang, Yuwei Liang, Shanshan Li, Yiping Xia, Liangsheng Zhang, Xiuyun Wang

**Affiliations:** Genomics and Genetic Engineering Laboratory of Ornamental Plants, College of Agriculture and Biotechnology, Zhejiang University, No. 866 Yuhangtang Road, West Lake District, Hangzhou 310058, China; Genomics and Genetic Engineering Laboratory of Ornamental Plants, College of Agriculture and Biotechnology, Zhejiang University, No. 866 Yuhangtang Road, West Lake District, Hangzhou 310058, China; Genomics and Genetic Engineering Laboratory of Ornamental Plants, College of Agriculture and Biotechnology, Zhejiang University, No. 866 Yuhangtang Road, West Lake District, Hangzhou 310058, China; Genomics and Genetic Engineering Laboratory of Ornamental Plants, College of Agriculture and Biotechnology, Zhejiang University, No. 866 Yuhangtang Road, West Lake District, Hangzhou 310058, China; Genomics and Genetic Engineering Laboratory of Ornamental Plants, College of Agriculture and Biotechnology, Zhejiang University, No. 866 Yuhangtang Road, West Lake District, Hangzhou 310058, China; Genomics and Genetic Engineering Laboratory of Ornamental Plants, College of Agriculture and Biotechnology, Zhejiang University, No. 866 Yuhangtang Road, West Lake District, Hangzhou 310058, China; Yazhouwan National Laboratory, No. 8 Huanjin Road, Yazhou District, Sanya 572024, China; Genomics and Genetic Engineering Laboratory of Ornamental Plants, College of Agriculture and Biotechnology, Zhejiang University, No. 866 Yuhangtang Road, West Lake District, Hangzhou 310058, China

## Abstract

Angiosperms are prolific producers of structurally diverse terpenes, which are essential for plant defense responses, as well as the formation of floral scents, fruit flavors, and medicinal constituents. Terpene synthase genes (TPSs) play crucial roles in the biosynthesis of terpenes. This study specifically focuses on the catalytic products of 222 functionally characterized TPSs in 24 angiosperms, which mainly comprise monoterpenes, sesquiterpenes, diterpenes, and sesterterpene. Our systematic analysis of these TPSs uncovered a significant expansion of the angiosperm-specific TPS-a, b, and g subfamilies in comparison to the TPS-e/f and c subfamilies. The expanded subfamilies can be further partitioned into distinct branches, within which considerable functional innovation and diversification have been observed. Numerous TPSs exhibit bifunctional or even trifunctional activities *in vitro*, yet they exhibit only a single activity *in vivo*, which may be largely determined by their inherent properties, subcellular localization, and the availabilities of endogenous substrates. Additionally, we explored the biological functions of terpenes in various organs and tissues of angiosperms. We propose that the expansion and functional divergence of TPSs contribute to the adaptability and diversity of angiosperms, facilitating the production of a broad spectrum of terpenes that enable diverse interactions with the environment and other organisms. Our findings provide a foundation for comprehending the correlation between the evolutionary features of TPSs and the diversity of terpenes in angiosperms, which is significant for terpene biosynthesis research.

## Introduction

Angiosperms, commonly known as flowering plants, overwhelmingly dominate the realm of terrestrial flora, representing nearly 90% of Earth’s botanical diversity [1, 2]. With a staggering number exceeding 350 000 extant species (http://www.theplantlist.org/), these plants have successfully colonized a vast array of biomes and ecosystems, showcasing remarkable adaptability and resilience [1]. To cope with ever-changing surroundings, plants have evolved an array of mechanisms to interact with the environment, including the emission of volatile metabolites from various organs such as flowers, leaves, and fruits [3]. Among these, terpenes, synthesized through specialized biochemical pathways, play a crucial role in ecological and physiological interactions.

Terpenes, also known as terpenoids, constitute the most extensive and structurally varied category of natural compounds [4]. Isopentenyl diphosphate (IPP) and dimethylallyl diphosphate (DMAPP), produced via the mevalonate (MVA) pathway in the cytosol or through the 2-C-methyl-D-erythritol 4-phosphate (MEP) pathway in plastids, serve as the universal precursor for all varieties of terpenes [5]. Moreover, the vast majority of terpenoids, which are often lineage-specific or even confined to individual species and thus designated as specialized terpenoids, are involved in a multitude of plant interactions with the environment and other organisms [6, 7]. These specialized terpenoids fulfill a spectrum of biological roles, including the attraction of pollinators and the resistance to pests and diseases [8]. In contrast, a limited number of terpenoids, such as phytohormones, pigments, sterols, plastoquinone, and ubiquinone, are recognized as primary terpenoid metabolites and found in all or nearly all plants that are essential for regulating growth and development [7, 9]. Terpenoids represent a ubiquitous class of compounds found in all organisms, widely synthesized by plants, animals, bacteria, and fungi [10]. Nonetheless, green plants, particularly angiosperms, possess a richer abundance of terpenoids compared to other living organisms [7]. To date, researchers have identified over 80 000 terpene metabolites from different living organisms [11]. Within this collection, the number of specialized plant terpenes with known structures numbers in the tens of thousands [7]. Notably, monoterpenes, a subset of specialized plant terpenes, have garnered significant research interest in recent years due to their remarkable chemical diversity in angiosperms [12]. Monoterpenes, along with other specialized terpenoids, find extensive application in the pharmaceutical, food, and cosmetic industries, including diterpene drugs like taxol and artemisinin, as well as the monoterpene limonene and the sesquiterpene valencene, which are major components of citrus oils [13, 14].

The biosynthesis of terpenes involves not only terpene synthases (TPSs)—the pivotal enzymes that are accountable for the diversity of plant terpene skeletons—but also a suite of downstream terpene-modifying enzymes, including cytochrome P450 monooxygenases (P450s), dehydrogenases, reductases, transferases, and isomerases, which are essential for the ultimate formation of functionalized terpenes [12, 15]. All full-length TPS proteins are marked by two conserved domains: an N-terminal domain and a C-terminal domain, identified by Pfam IDs PF01397 and PF03936, respectively [16], as annotated in the Pfam database (http://pfam.xfam.org/) [17]. TPS can synthesize a variety of terpenes from different substrates, such as DMAPP, geranyl diphosphate (GPP), farnesyl diphosphate (FPP), geranylgeranyl diphosphate (GGPP), and geranylfarnesyl diphosphate (GFPP), which serve as precursors for the biosynthesis of hemiterpenes (C5), monoterpenes (C10), sesquiterpenes (C15), diterpenes (C20), and sesterterpenes (C25), respectively [18, 19]. Furthermore, when two C15 FPP and two C20 GGPP molecules sequentially undergo “head-to-head” condensations, resulting in precursors of triterpenes (C30) and tetraterpenes (C40), correspondingly [9, 20]. In addition, a distinctive terpene biosynthetic pathway was discerned in roses, where a Nudix hydrolase, *RhNUDX1* catalyzed GPP in the cytosol to ultimately produce geraniol [21]. The origin of GPP in the cytosol has been a subject of extensive debate, with some evidence indicating that in certain plants, it is transported from the plastids [22, 23]. However, the universality of this mechanism across plants remains to be fully elucidated.

**Table 1:** The number of main terpenoids produced by the functionally characterized TPSs in the seven most studied angiosperms

	TPSs	Monoterpenes	Sesquiterpenes	Diterpenes	Sesterterpenes
*Arabidopsis thaliana*	26	8	7	6	8
*Solanum lycopersicum*	34	8	19	4	0
*Cannabis sativa*	28	8	12	0	0
*Vitis vinifera*	30	9	22	1	0
*Cucumis sativus*	17	7	8	1	0
*Oryza sativa*	24	5	11	12	0
*Wurfbainia villosa*	18	10	12	1	0

Despite comprehensive research into the terpenoid metabolites generated by functionally characterized TPSs across various angiosperms, particularly in model plants such as Arabidopsis (*Arabidopsis thaliana*), tomato (*Solanum lycopersicum*), and rice (*Oryza sativa*), the evolutionary mechanisms that underpin the functional diversity of TPSs are still not well understood. In this study, we collated data on the metabolites produced by 222 functionally characterized TPSs in 24 angiosperm species, predominantly encompassing monoterpenes, sesquiterpenes, diterpenes, and sesterterpene. Following this, we performed an evolutionary analysis of these functionally characterized TPSs to reveal their evolutionary features, shedding light on the genetic factors driving the functional diversity of TPSs within angiosperms. Additionally, a thorough examination of the angiosperm TPS gene family uncovered the functional diversification of genes along different evolutionary clades and subclades. Finally, we described the biological functions of terpenes in various organs and tissues of angiosperms. This study offers insights into the evolutionary patterns and functional differences of TPSs in angiosperms and provides a roadmap for future inquiries into plant terpene metabolism.

## Results and discussions

### Diversity of terpenes synthesized by functionally characterized TPSs in angiosperms

In our study, we sourced 222 functionally characterized TPSs from 24 angiosperms (including 15 eudicots, and 9 monocots; Supplementary Data Tables S1), all of which have been experimentally validated and their substrates are well-defined (Supplementary Data Tables S2–S6). Our analysis revealed that 120 TPSs utilized GPP or neryl diphosphate (NPP) as substrates to produce monoterpenes, with linalool, myrcene, (*E*)-*β*-ocimene, geraniol, and (+)-limonene being the most commonly synthesized. Meanwhile, 121 TPSs catalyzed *trans*/*cis*-farnesyl diphosphate (*E,E*-FPP/*Z,Z*-FPP) to form sesquiterpenes, with (*E*)-nerolidol, (*E*)-*β*-farnesene, nerolidol, *β*-bisabolene, (*E,E*)-*α*-farnesene, and *α*-humulene as the predominant products. Among the class II diterpene synthases (diTPSs), 8 copalyl diphosphate synthases (CPSs) used GGPP to synthesize copalyl diphosphate (CPP). Furthermore, 3 kaurene synthases (KSs) and 8 kaurene synthases-like (KSLs), classified as class I diTPSs, catalyzed the transformation of CPP into *ent*-kaurene, which is involved in gibberellin (GA) biosynthesis, and some intermediates acting as the precursors to labdane-related diterpenoids, respectively. Moreover, 15 other diTPSs utilized GGPP or nerylneryl diphosphate (NNPP) to produce a variety of diterpenes, including geranyl linalool, lycosantalene, and dolathaliatriene. In several Brassicaceae plants, 11 TPSs were found to catalyze the synthesis of various sesterterpenes from GFPP, such as (−)-*ent*-quiannulatene, (−)-variculatriene A, and (+)-astallatene. Although extensive functional validations of TPSs have been conducted in Arabidopsis, tomato, grape (*Vitis vinifera*), cannabis (*Cannabis sativa*), rice, etc. (Table 1), the majority of angiosperms have only been investigated for a limited number of TPSs. Notably, tomato boasts the highest number of functionally characterized TPSs among these angiosperms, with a total of 34 confirmed through *in vitro* enzyme activity characterization [24]. These TPSs in tomato are mainly responsible for the synthesis of 8 monoterpenes, 19 sesquiterpenes, and 4 diterpenes (Table 1).

**Supplementary Data Table S1:**
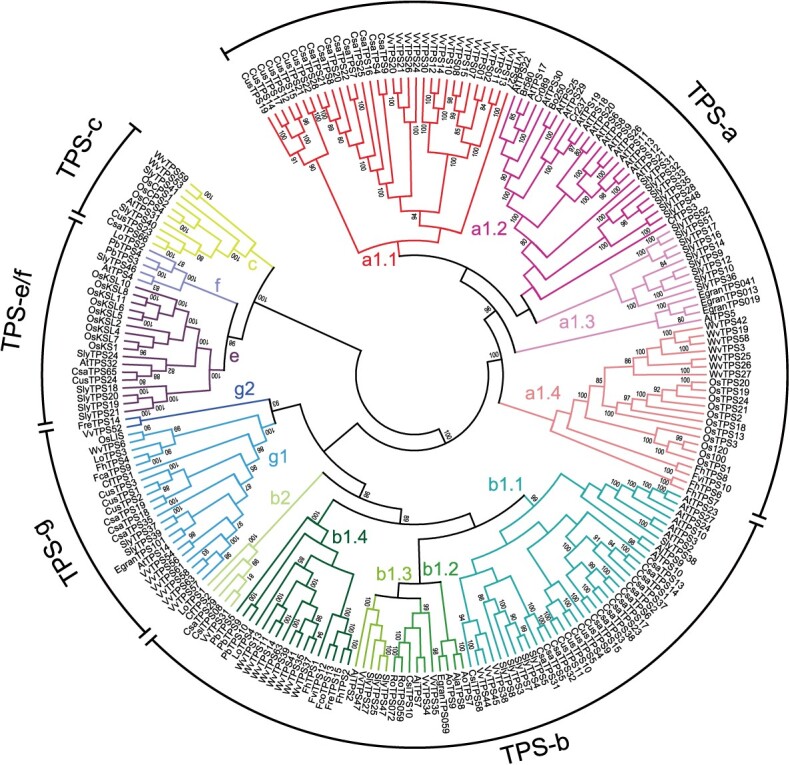


**Supplementary Data Table S2:**
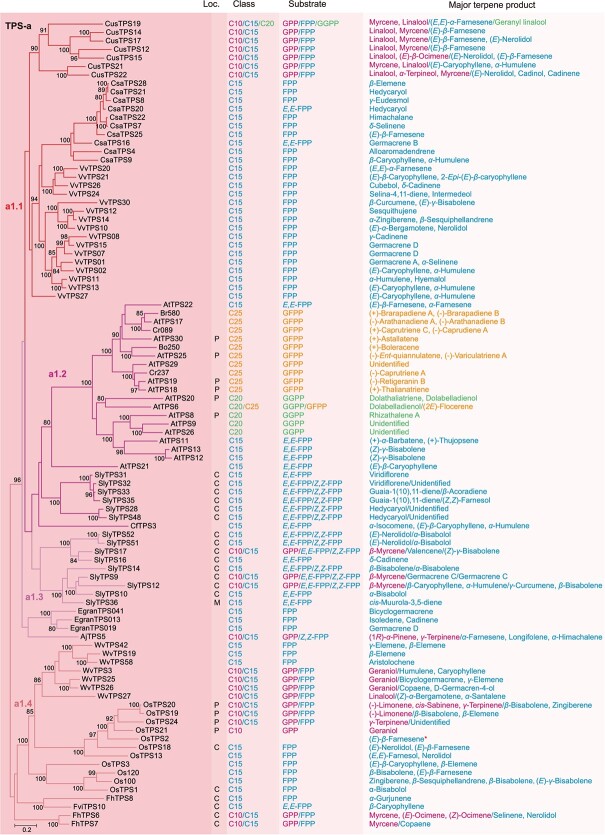


**Supplementary Data Table S3:**
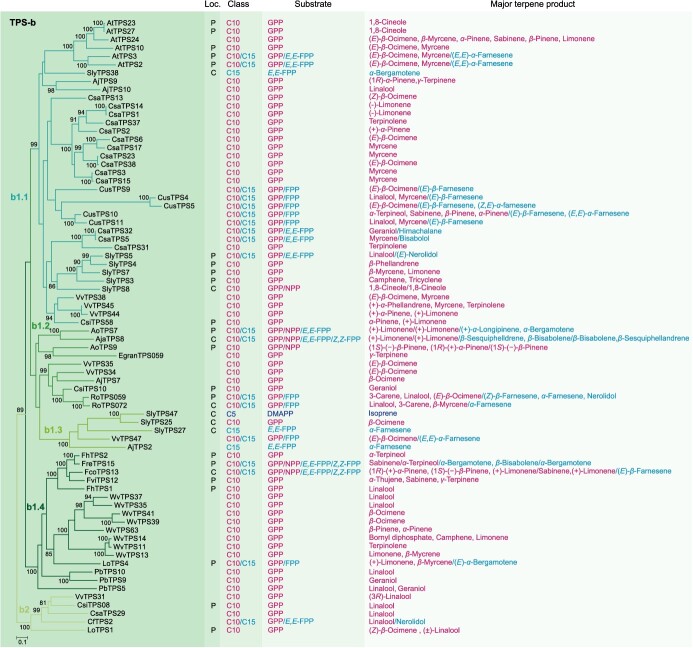


**Supplementary Data Table S4:**
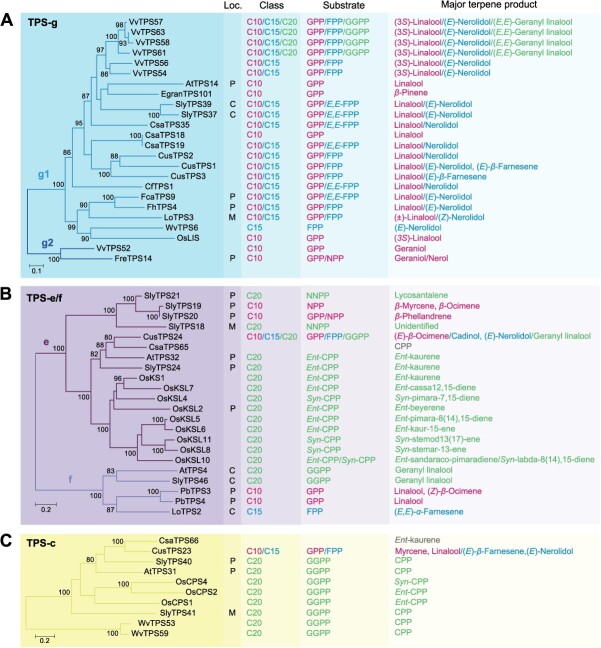


**Supplementary Data Table S5:**
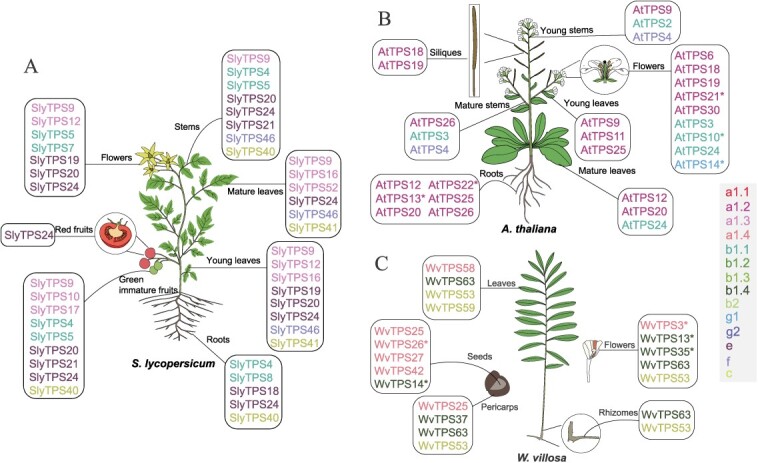


**Supplementary Data Table S6:**
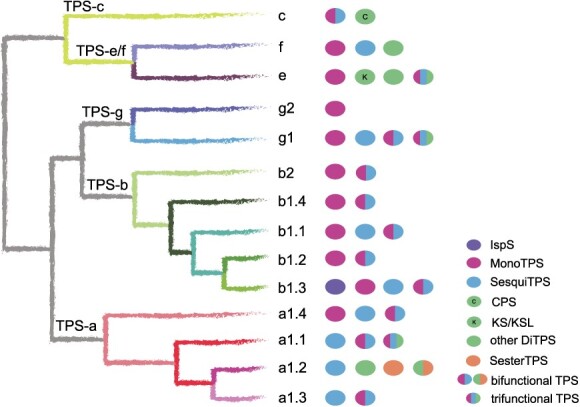


### Phylogenetic analysis reveals the significant expansion and divergence of TPS-a, b, and g genes

The rich variety of terpene structures found in plants is attributed to the evolution of new genes that encode new terpene synthases [7]. Gene duplication plays a pivotal role in this process, facilitating the acquisition of genetic innovation and contributing significantly to the expansion of TPSs within angiosperms. A recent study on the mechanism of monoterpene synthases (MTSs) in generating the chemical diversity of monoterpenes suggests that by aligning the proposed active-site residues in angiosperm MTSs, conserved amino acids, motifs, or regions may be engaged in the fundamental aspects of catalysis, whereas the variable ones may be related to the formation of different monoterpenes [12]. Comprehensive research indicates that the TPS gene family is typically of moderate size in angiosperms, with the number of genes varying from a handful to more than a hundred in the genomes of sequenced plants [18]. This genetic wealth offers a vast platform for the ongoing evolution of new terpenoids. Previous phylogenetic studies have classified the TPS gene family into seven subfamilies: TPS-a, b, c, e/f, g, d, and h, of which only the first five are present in angiosperms [18]. TPS-a, b, and g are angiosperm-specific subfamilies, whereas TPS-e/f and c exhibit a more ancient evolutionary history [25].

**Figure 1:**
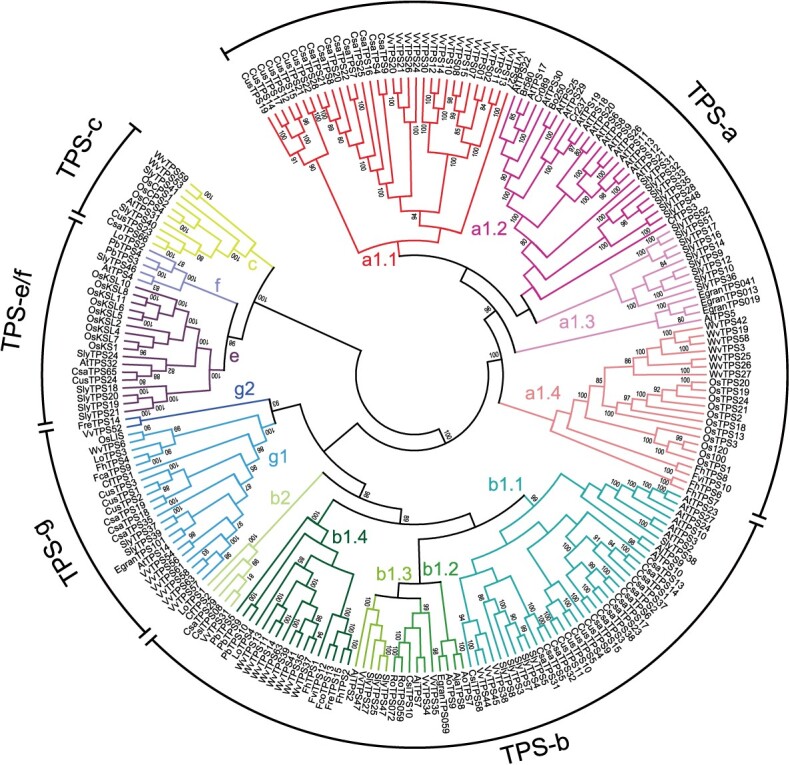
A rooted phylogenetic tree of functionally characterized TPSs in angiosperms. The tree encompasses TPS protein sequences from 15 eudicots and 9 monocots. The five previously defined TPS subfamilies (a, b, g, e/f, and c) in angiosperms are indicated and branches are color-coded based on different clades and subclades. The TPS-a subfamily contained only one clade (a1), while the TPS-b and g subfamilies were classified into two major clades (b1 and b2; g1 and g2). The a1 and b1 clades were further divided into four subclades (a1.1, a1.2, a1.3, and a1.4; b1.1, b1.2, b1.3, and b1.4).

To gain insights into the evolutionary features of TPSs in angiosperms and elucidate the functional diversity of the TPS gene family, we undertook an in-depth evolutionary analysis using 222 functionally characterized TPSs from 24 angiosperms. These TPSs encompass all five TPS subfamilies identified in angiosperms, with a notable predominance of genes within the TPS-a and TPS-b subfamilies. Subsequently, we utilized the protein sequences of these TPSs to construct a maximum likelihood (ML) tree (Fig. 1). The phylogenetic analysis uncovered substantial variation in evolutionary patterns among the different subfamilies, highlighting the dynamic nature of TPS gene evolution in angiosperms.

**Figure 2:**
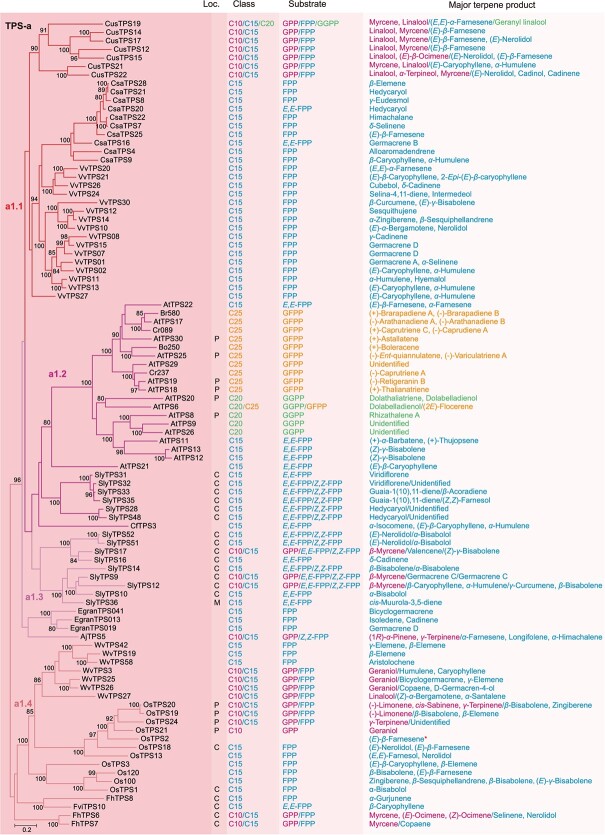
Phylogenetic analysis and functional characteristics of TPS-a genes in angiosperms. In the left panel, a phylogenetic tree of the TPS-a genes is depicted, utilizing the ML method. The right four panels show the experimentally proved subcellular localization (Loc.), product class, specific substrate, and major terpene product. The font colors of the compounds C10 (monoterpene), C15 (sesquiterpene), C20 (diterpene), and C25 (sesterterpene) and their corresponding specific substrates as well as major products are indicated by red, blue, green, and orange, respectively. The *in vivo* enzymatic product of *OsTPS2* is marked by an asterisk. GPP, geranyl diphosphate; FPP, farnesyl diphosphate; *E,E*-FPP/*Z,Z*-FPP, *trans*/*cis*-farnesyl diphosphate; GGPP, geranylgeranyl diphosphate; GFPP, geranylfarnesyl diphosphate; C, cytosolic localization; P, plastidic localization; M, mitochondrial localization.

**Figure 3:**
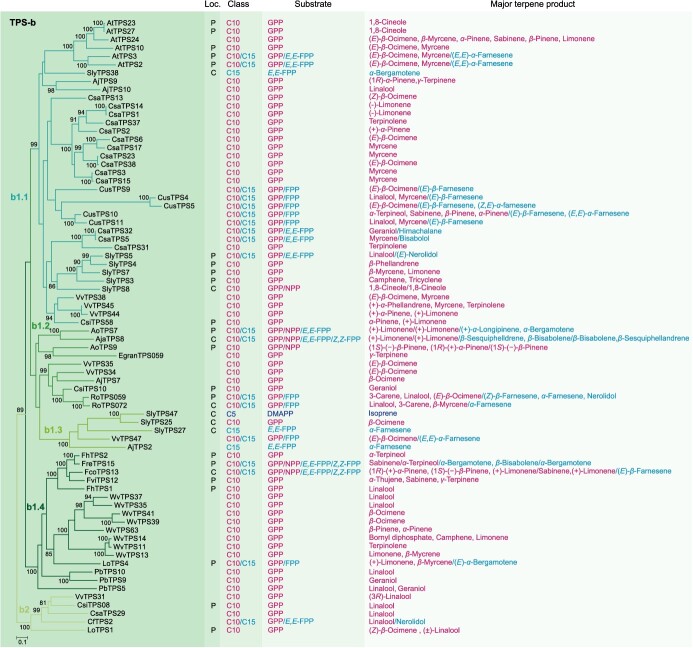
Phylogenetic analysis and functional characteristics of TPS-b genes in angiosperms. The left panel shows the ML tree of the TPS-b genes. The right four panels display the experimentally proved subcellular localization (Loc.), product class, specific substrate, and major terpene product. The font colors of the compounds C5 (hemiterpene), C10 (monoterpene), and C15 (sesquiterpene) and their corresponding specific substrates as well as major products are indicated by purple, red, and blue, respectively. DMAPP, dimethylallyl diphosphate; GPP, geranyl diphosphate; NPP, neryl diphosphate; FPP, farnesyl diphosphate; *E,E*-FPP/*Z,Z*-FPP, *trans*/*cis*-farnesyl diphosphate; C, cytosolic localization; P, plastidic localization.

**Figure 4:**
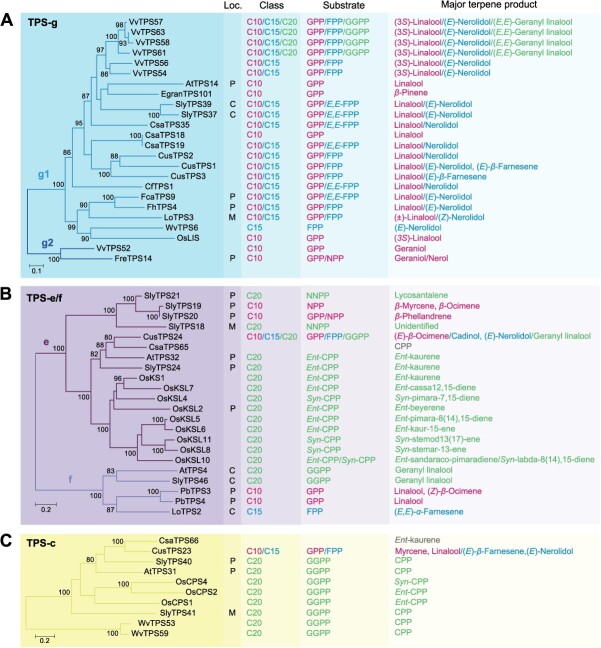
Phylogenetic analysis and functional characteristics of TPS-g, e/f, and c genes in angiosperms. (A) Evolution and function of TPS-g genes. (B) Evolution and function of TPS-e/f genes. (C) Evolution and function of TPS-c genes. The left panel shows three ML trees of the TPS-g, e/f, and c subfamilies, respectively. The right four panels display the experimentally proved subcellular localization (Loc.), product class, specific substrate, and major terpene product. The font colors of the compounds C10 (monoterpene), C15 (sesquiterpene), and C20 (diterpene) and their corresponding specific substrates as well as major products are indicated by red, blue, and green, respectively. Products shown in grey indicate putative products, not validated. GPP, geranyl diphosphate; NPP, neryl diphosphate; FPP, farnesyl diphosphate; *E,E*-FPP, *trans*-farnesyl diphosphate; GGPP, geranylgeranyl diphosphate; NNPP, nerylneryl diphosphate; CPP, copalyl diphosphate; C, cytosolic localization; P, plastidic localization; M, mitochondrial localization.

Our analysis revealed that the TPS-a, b, and g subfamilies have experienced a rapid expansion in angiosperms, in contrast to the more conserved TPS-e/f and c subfamilies (Fig. 1). This observation aligns with previous research [12, 26]. The TPS-a subfamily was comprised of 94 TPSs, with monocotyledon and dicotyledon TPSs forming distinct clusters (Fig. 2). It only contained one single major clade (a1), which was further divided into four subclades (a1.1, a1.2, a1.3, and a1.4) (Fig. 2). The a1.1, a1.2, and a1.3 subclades were associated with eudicots, while the a1.4 subclade was exclusive to monocots. Notably, the TPS-a subfamily exhibited a pronounced species-clustered phenomenon, suggesting that species-specific expansion may have taken place. For example, the TPSs of grape, cucumber (*Cucumis sativus*), and cannabis were clustered within the a1.1 subclade, with intraspecific TPSs grouping closely together. Similarly, the TPSs of rice and *Wurfbainia villosa* were clustered within the a1.4 subclade, with intraspecific TPS genes forming tight clusters. Furthermore, we observed lineage-specific expansions in the TPS-a subfamily, such as the Brassicaceae-specific expansion of TPS genes within the a1.1 subclade (Fig. 2). Current understanding suggests that the TPS-b and g subfamilies are closely associated with the TPS-a subfamily while exhibiting significant divergence from the other TPS subfamilies [27]. The TPS-b subfamily, acting as the sister group to TPS-g, has a more intricate evolutionary history and comprises two major clades (b1 and b2), encompassing a total of 74 TPSs (Fig. 3). Notably, the b1 clade was found to be more abundant than the b2 clade, which was relatively conserved. The b1 clade was further divided into four subclades, mirroring the pattern observed in the a1 clade, with the b1.1, b1.2, and b1.3 subclades representing eudicot groups, and the b1.4 representing the monocot group (Fig. 3). The TPS-g subfamily included 24 TPSs, similar to TPS-b, and was structured around two main clades, with the g1 clade comprising 22 members, outnumbering the g2 clade, which had only 2 members (Fig. 4A). The TPS-e/f and c subfamilies were more conserved, consisting of 21 and 9 TPSs, respectively (Fig. 4B and C).

### Functional diversity of TPSs

TPSs have undergone species-specific duplication and divergence throughout their evolutionary history, leading to the development of diverse functions among TPS members across different subfamilies. In essence, each subfamily plays a role in influencing the synthesis of various terpenoid compounds [28]. The TPS-c and e/f subfamilies are not only involved in the synthesis of primary metabolites but also contribute to the production of a wide array of specialized metabolites. Conversely, the TPS-a, b, and g subfamilies predominantly catalyze the formation of specialized metabolites [18, 29].

In general, the TPS-a and b subfamilies are responsible for the production of sesquiterpenes and monoterpenes in the cytoplasm and plastids, respectively [18]. Closely correlated with TPS-b but lacking the conserved R(R)X8W motif necessary for the start of the isomerization cyclization reaction [30], the members of the TPS-g subfamily primarily synthesize acyclic products [18]. The TPS-e/f genes, found in vascular plants, predominantly synthesize *ent-*kaurene, as well as other diterpenes, monoterpenes, and sesquiterpenes, whereas the TPS-c genes, present in land plants, are mainly responsible for the biosynthesis of CPP and other diterpenes [18, 28]. However, recent years have seen an increasing complexity in the evolutionary trajectory of plant terpene biosynthetic pathways, marked by the emergence of numerous new enzymes and substrates [20]. Consequently, the functions attributed to TPSs are becoming more complex than previously thought. It is speculated that each of the five subfamilies in angiosperms, along with their respective clades and subclades, likely plays distinct roles in terpene biosynthesis, contributing to the diverse array of terpenes produced.

#### Angiosperm-specific TPS-a, b, and g genes are responsible for the biosynthesis of specialized metabolites

Within the TPS-a subfamily, the majority of members specialized in producing sesquiterpenes using FPP as a substrate, while some TPSs are capable of synthesizing monoterpenes, diterpenes, or sesterterpenes (Fig. 2; Supplementary Data Table S2). Subcellular localization studies have revealed that most TPS-a genes are cytosolic, with a smaller number localized in the plastids and mitochondria. Notably, 14 out of 15 functionally characterized TPS-a genes in tomato are cytosolic, with one gene localized to the mitochondria [24, 31]. In contrast, all six examined TPS-a genes in Arabidopsis are plastid-localized [32–36]. Rice exhibits a different pattern, with two TPS-a genes in the cytosol and four in the plastids [37–41]. In the *Freesia* genus, including *Freesia × hybrida* and *Freesia viridis*, four TPS-a genes have been found in the cytosol [42, 43]. Focusing on the a1.1 subclade, all functionally characterized TPSs are capable of synthesizing sesquiterpenes from FPP, such as germacrene D, (*E*)-*β*-farnesene, (*E*)-nerolidol, and *α*-humulene. *In vitro* enzyme activity studies have shown that 10 a1.1 subclade genes from *C. sativa* and 16 from *V. vinifera* both produce sesquiterpenes from FPP [44–47]. Remarkably, certain bifunctional TPS-a genes in *C. sativus* can use both GPP and FPP as substrates to synthesize monoterpene and sesquiterpene *in vitro*, respectively. For instance, three adjacent TPSs within this subclade, *CusTPS12*, *14*, and *17*, have been shown to catalyze the formation of several monoterpenoids from GPP, including linalool and myrcene, and predominantly (*E*)-*β*-farnesene from FPP [48]. Additionally, *CusTPS19* is a standout example of the trifunctional TPS gene, with activities extending to the production of monoterpenes and sesquiterpenes from GPP and FPP, and a diterpene, geranyl linalool from GGPP [48, 49].

In the a1.2 subclade, the functionally characterized TPSs are primarily from tomato and Brassicaceae. Both TPSs of the a1.2 and a1.3 subclades in tomato synthesized sesquiterpenes from *E,E*-FPP, and some of them also exhibit activity with *Z,Z*-FPP. The situation of TPS functions in Arabidopsis is more complex. *AtTPS11*, *12*, *13*, and *22* produced sesquiterpenes using *E,E*-FPP [33, 50, 51]. *AtTPS8*, *9*, *20*, and *26* used GGPP to synthesize diterpenes, such as rhizathalene A, dolabelladienol, and dolathaliatriene [32, 33]. *AtTPS17*, *18*, *19*, *25*, *29*, and *30* were involved in the formation of sesterterpenes from GFPP, including (−)-arathanadiene A, (−)-*ent*-quiannulatene, (−)-variculatriene A, and (+)-astallatene [35, 36, 52, 53]. *AtTPS6* was a bifunctional di−/sesterterpene synthase, producing dolabelladienol from GGPP and (*2E*)-flocerene from GFPP [33, 53, 54]. Additionally, *Cr237* and *089* in *Capsella rubella*, *Bo250* in *Brassica oleracea*, and *Br580* in *Brassica rapa* produced sesterterpenes from GFPP, including (+)-boleracene, (+)-brarapadiene A, (+)-caprutriene C, and (−)-caprutriene A [34, 52].

In the a1.3 subclade, similar to the members of the a1.1 subclade, all functionally characterized TPSs encode enzymes that catalyze the formation of sesquiterpenes from FPP. *SlyTPS9*, *12*, and *17* in tomato were multiproduct enzymes that used GPP, *E,E*-FPP, and *Z,Z*-FPP as substrates to synthesize monoterpene and sesquiterpene, respectively [55–57]. An interesting case is *AjTPS5* from *Albizia julibrissin*, which is a bifunctional TPS gene primarily producing two monoterpenes, (1*R*)-*α*-pinene and *γ*-terpinene from GPP, and three sesquiterpenes, *α*-farnesene, longifolene, and *α*-himachalene from *Z,Z*-FPP [58]. All TPSs of the a1.4 subclade are known to produce sesquiterpenes using FPP as the substrate, with the exception of *OsTPS21* in *O. sativa*, which exhibits activity only with GPP, predominantly mostly producing geraniol [41]. Additionally, some bifunctional TPSs in rice, *W. villosa*, and *F. hybrida* have been found to use GPP for monoterpene production [38, 40, 43, 59].

In the TPS-b subfamily, the majority of members can produce monoterpenes using GPP or NPP, with certain TPSs capable of synthesizing sesquiterpenes from FPP (Fig. 3; Supplementary Data  Table S3). Among the 29 TPSs in this subfamily that have been examined for subcellular localization, 21 were found in the plastids and eight in the cytosol. Within the b1.1 subclade, most TPSs are involved in monoterpene synthesis using GPP, with the exception of *SlyTPS38*, which was localized in the cytosol and primarily produced *α*-bergamotene from *E,E*-FPP [31]. Another cytosol-localized gene, *SlyTPS8*, stands out for its activity with both GPP and NPP, primarily catalyzing the formation of 1,8-cineole [31]. Additionally, many bifunctional mono−/sesquiterpene synthases have been identified in *C. sativa*, cucumber, and Arabidopsis. All TPSs in the b1.2 clade are capable of producing monoterpenes using GPP or NPP. Notably, *RoTPS059* and *072* in *Rhododendron ovatum*, *AoTPS7* in *Aquilegia oxysepala*, and *AjaTPS8* in *Aquilegia japonica*, are bifunctional TPSs that synthesized monoterpenes and sesquiterpenes [60, 61]. The b1.3 subclade comprises five genes, including three from tomato (*SlyTPS25*, *27*, and *47*), which exhibit distinct functions despite being cytosol-localized. *SlyTPS47*, initially showing no activity with tested substrates (GPP, NPP, *E,E*-FPP, *Z,Z*-FPP, and GGPP), was later found to catalyze isoprene from DMAPP [24]. *SlyTPS25* is specific to GPP, primarily producing *β*-ocimene, while *SlyTPS27* used *E,E*-FPP to synthesize *α*-farnesene [24]. In a study on grape TPS functions, *VvTPS47* was active with both GPP and FPP, producing (*E*)-*β*-ocimene and (*E,E*)-*α*-farnesene, respectively [44]. *AjTPS2*, similar to *SlyTPS27*, primarily synthesized *α*-farnesene from *E,E*-FPP [58]. Members of the b1.4 subclade catalyzed the formation of monoterpenes using GPP or NPP, with some also showing activity with FPP to produce sesquiterpenes, such as *LoTPS4* in *Lilium* ‘Siberia’ [62], *FreTPS15* in *Freesia refracta* and *FcoTPS13* in *Freesia corymbose* [42]. TPSs of the b2 subclade primarily synthesized monoterpenes using GPP. *CfTPS2* in *Clematis florida* is a bifunctional enzyme with both GPP and *E,E*-FPP activities, primarily producing linalool and nerolidol, respectively [63].

The TPS-g subfamily is characterized by the prevalence of bifunctional or trifunctional TPSs, exhibiting multiple substrate activities *in vitro* (Fig. 4A; Supplementary Data Table S4). Subcellular localization studies have revealed that TPS-g genes were located in the cytosol, plastids, or mitochondria. Within the g1 clade, several TPSs are specialized in the synthesis of monoterpenes using GPP as the substrate. For instance, *AtTPS14*, *CsaTPS18* in *C. sativa*, *EgranTPS101* in *Eucalyptus grandis*, and *OsLIS* in rice primarily produce linalool, (3*S*)-linalool, and *β*-pinene, respectively [8, 47, 64–66]. *WvTPS6* in *W. villosa* is unique in its preference for *E,E*-FPP as the substrate to produce (*E*)-nerolidol [59]. The remaining TPSs in the g1 clade are involved in the synthesis of both monoterpenes from GPP and sesquiterpenes from FPP. Notably, four TPSs in grape (*VvTPS57*, *58*, *61*, and *63*) share similar functions, being active with GGPP to produce (*E,E*)-geranyl linalool, in addition to synthesizing (3*S*)-linalool from GPP and (*E*)-nerolidol from FPP [44]. The g2 subclade, comprising only two genes, is dedicated to the formation of geraniol from GPP. Furthermore, the plastid-localized *FreTPS14* also produced nerol using NPP as a substrate [42].

#### TPS-e/f and c genes primarily encode CPS and KS(L)

In addition to synthesizing a diverse array of specialized metabolites, members of the TPS-e/f and c subfamilies are also capable of catalyzing the synthesis of *ent*-kaurene (Fig. 4B and C; Supplementary Data Tables S5 and S6). This compound is essential for the production of plant gibberellins and related phytohormones, which play crucial roles in plant growth and development [29]. The biosynthesis of *ent*-kaurene involves the conversion of GGPP into *ent*-CPP by CPSs, followed by the action of KSs to transform *ent*-CPP into *ent*-kaurene [67]. Subcellular localization studies have indicated that the majority of TPS-e/f and c genes are localized in the plastids, with a smaller number found in the cytosol and mitochondria. This localization pattern suggests that these subfamilies play a significant role in the plastidic metabolism of terpenes, particularly in the synthesis of gibberellins and other related compounds.

Three TPS-e genes (*SlyTPS24*, *AtTPS32*, and *OsKS1*) encoded KSs that catalyzed the formation of *ent*-kaurene [24, 68–70]. In rice, *OsKSL2*, *4*, *5*, *6*, *7*, *8*, *10*, and *11* encode KSLs that catalyze the conversion of *ent-*CPP or *syn-*CPP to produce intermediates that serve as precursors to labdane-related diterpenoids which are shown to function as terpenoid phytoalexins [71–78]. Notably, *OsKSL10* exhibits dual activity with both *ent-*CPP and *syn-*CPP as substrates, producing *ent-*sandaraco-pimaradiene and *syn*-labda-8(14),15-diene, respectively [71, 78]. Furthermore, the mitochondria-localized *SlyTPS18* and the plastid-localized *SlyTPS21* are genuine diterpene synthases, using NNPP to produce lycosantalene and an unidentified diterpene, respectively [24, 79]. Another two plastid-localized genes *SlyTPS19* and *20* catalyzed the formation of several monoterpenes (*β*-myrcene, *β*-ocimene, and *β*-phellandrene) from GPP or NPP [24, 79, 80]. *CusTPS24*, a trifunctional TPS gene, showed activities with GPP, *E,E*-FPP, and GGPP, primarily producing (*E*)-*β*-ocimene, cadinol, and geranyl linalool, respectively [48]. Two cytosol-localized TPS-f genes (*AtTPS4* and *SlyTPS46*) used GGPP to produce geranyl linalool, whereas *LoTPS2*, also cytosol-localized, only had activity with FPP to produce (*E,E*)-*α*-farnesene [24, 62, 81, 82]. Additionally, the plastid-localized *PbTPS3* and *4* in *Phalaenopsis bellina* primarily synthesized linalool from GPP [83].

In addition to *CusTPS23* in *C. sativus*, which produced myrcene and linalool from GPP, and (*E*)-*β*-farnesene and (*E*)-nerolidol from FPP [48], the majority of members in the TPS-c subfamily are involved in catalyzing the formation of CPP. *CsaTPS66* in *C. sativa*, while annotated as a KS, has yet to have its function fully validated, making it a subject for future research [47].

#### Functional differences of TPSs between *in vivo* and *in vitro* due to subcellular compartment

Many TPSs display bifunctional or trifunctional activities with multiple substrates *in vitro*. However, *in vivo*, each TPS gene is located within a specific subcellular compartment, enabling the utilization of specific substrates to produce specific terpenes. Consequently, the function of TPSs may be influenced by their inherent characteristics and subcellular localization.

A recent study on the floral scent evolution in azalea revealed that two functionally characterized TPSs of the TPS-b subfamily in *R. ovatum*, *RoTPS059* and *072*, exhibited activities with both GPP and FPP *in vitro*, producing several monoterpenes and sesquiterpenes, respectively [60]. However, *in vivo*, the plastid-localized *RoTPS059* primarily catalyzed the synthesis of 3-carene, whereas the cytosol-localized *RoTPS072* mainly produced *α*-farnesene [60]. This indicates that subcellular localization plays a critical role in determining the substrate preference and product profile of TPSs. Additionally, a previous study on *W. villosa* TPSs (*WvTPS3*, *25*, and *26*) from the TPS-a subfamily found that all major products were geraniol when GPP was used as the substrate [59]. However, when an equiproportional mixture of GPP and FPP was used, *WvTPS58* only synthesized several sesquiterpenes, whereas *WvTPS25* and *26* catalyzed the synthesis of both monoterpenes and sesquiterpenes, with a higher proportion of sesquiterpenes [59]. This suggested that although *WvTPS25* and *26* showed activities with both GPP and FPP *in vitro*, they displayed a higher substrate preference towards FPP compared to GPP, likely influenced by their subcellular localization and the availability of endogenous substrates.

### Biological functions of terpenes catalyzed by diverse TPSs in various organs

Angiosperms produce a variety of terpenes in significant quantities across various organs, with each terpene being catalyzed by corresponding enzymes and performing distinct biological functions. For instance, in flowers, terpenes contribute to the creation of pleasant floral scents, which are essential for attracting pollinators. In fruits, these compounds enhance the aroma and flavor, attracting fruit-eating animals and facilitating seed dispersal. Furthermore, certain terpenes in plant organs and tissues exhibit both biotic and abiotic defense functions. Notably, many terpenes are not confined to a single function; they are often present in multiple organs and can play overlapping roles in various biological aspects [84].

**Figure 5:**
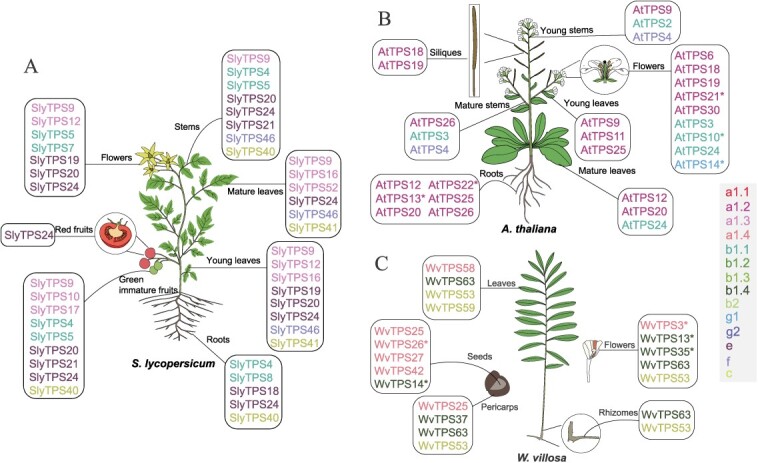
Expression of TPSs in different organs of two model angiosperms and one well-known medicinal angiosperm. (A) Expression of tomato TPSs in different organs. (B) Expression of Arabidopsis TPSs in different organs. (C) Expression of TPSs of *W. villosa* in different organs. The image of *W. villosa* was modified from that in a previous study [59]. The TPSs depicted in the figure show relatively high expression levels in different organs, with asterisks denoting organ-specific expression. The color of each TPS gene corresponds to different gene tree branches.

To gain a deeper understanding of the functional differences of TPSs across various organs, we conducted gene expression analysis of TPSs in two model species and one medicinal plant using public datasets. Our analysis of TPS expression profiles in these plants revealed that the composition and expression levels of TPS genes varied significantly across different organs (Fig. 5). For instance, in Arabidopsis, TPS genes that expressed in the root system primarily belong to the a1.2 subclade, whereas in flowers, genes were derived from the a1.2, b1.1, and g1 subclades (Fig. 5B) [36]. These genes from different branches may play diverse biological roles in various organs, underscoring the complexity and specificity of TPS function in different plant tissues.

Angiosperms are renowned for their attractive blooms, many of which exude delightful aromas. Some research has indicated that terpenes are often the primary constituents of floral scents in numerous angiosperms. Two members from the b1.2 subclade of TPSs, *RoTPS059* and *072*, produced 3-carene and *α*-farnesene, respectively, which are the main components affecting the floral scent of *R. ovatum* [60]. Similarly, *FviTPS10* in the a1.4 subclade and *FreTPS14* in the g2 clade produced *β*-caryophyllene and nerol, respectively, which are the main fragrance components of *F. viridis* and *F. refracta* flowers [42]. *SlyTPS5* of the b1.1 subclade was highly expressed in tomato fruits (Fig. 5A) and was responsible for the synthesis of linalool [85], which has been shown to enhance the flavor and aroma of tomato fruits [86, 87]. In addition, volatile terpenes released from flowers, fruits, and other organs of angiosperms play a crucial role in attracting pollinators and beneficial organisms. In kiwifruit, terpenes are believed to be important for attracting fruit-eating animals, thereby facilitating seed dispersal through the promotion of fruit flavor and aroma [84]. Notably, certain terpenes serve as medicinal ingredients. The seeds of *W. villosa*, a traditional Chinese medicinal plant, are abundant in volatile terpenoids, with bornyl acetate, borneol, camphor, limonene, and camphene being the major compounds [59]. *WvTPS14* in the b1.4 subclade, which exhibited seed-specific high expression, is responsible for the biosynthesis of these five terpenoids (Fig. 5C) [59].

When plants are attacked by herbivores or microorganisms, they may produce a significant amount of volatile terpenoids to directly attack these organisms or indirectly attract their enemies [88, 89]. A previous study demonstrated that *GhTPS12* in *Gossypium hirsutum* only produced (3*S*)-linalool *in vivo*, which was one of the most abundant volatile compounds induced by insect herbivory, primarily aphids, and moths, indicating its role in direct defense responses [90]. Similarly, the promoter activities of *CusTPS2* of the g1 clade, *CusTPS9* of the b1.1 subclade, and *CusTPS19* of the a1.1 subclade were induced in cucumber leaves upon infestation by *Tetranychus urticae*, *Frankliniella occidentalis*, and *Myzus persicae*, respectively, leading to the production of various volatile terpenoids as the defense against these herbivorous insects [48]. In plants of the Poaceae family, especially in rice, terpenes have been recognized for their critical role in plant defense mechanisms. Four members of the a1.4 subclade in rice, *OsTPS24*, *20*, *12*, and *18*, are responsible for producing the monoterpenes *γ*-terpinene, (*S*)-limonene, and geraniol, as well as the sesquiterpene (*E*)-nerolidol, respectively. These TPSs have shown significant antibacterial activity against *Xanthomonas oryzae* pv. oryzae (*Xoo*), the bacterium causing bacterial blight in rice [38–41]. Additionally, (*S*)-limonene also contributes to rice defense against *Magnaporthe oryzae*, the fungus responsible for rice blast disease [40]. Furthermore, many labdane-type diterpenoids, discovered in rice, act as antimicrobial phytoalexins, which are generally combined with volatile monoterpene and sesquiterpene defenses [91–94]. KSLs, typically found in the TPS-e clade, catalyze the generation of intermediates from CPP, which ultimately form labdane-related diterpenes under the action of cytochrome P450 monooxygenases, including phytocassanes, oryzalides, momilactones, and oryzalexins [94, 95]. For example, *OsKSL6* of the TPS-e clade produced *ent*-kaur-15-ene, the precursor to oryzalides A-C, which exhibit antibacterial activity against *Xoo* in rice [74, 96, 97].

## Conclusions and perspectives

**Figure 6:**
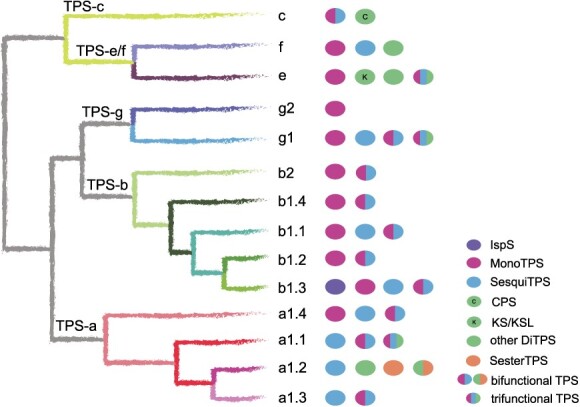
Evolutionary model and function of the TPS gene family in angiosperms. A proposed evolutionary model of TPSs is on the left. Ovals on the right show the functions of TPSs in different branches. Different colored ovals represent different types of TPS. Ovals containing two colors represent branches with bifunctional TPS, and ovals containing three colors represent branches with trifunctional TPS. IspS, isoprene synthase; MonoTPS, monoterpene synthase; SesquiTPS, sesquiterpene synthase; DiTPS, diterpene synthase; CPS, copalyl diphosphate synthase; KS, kaurene synthase; KSL, kaurene synthase-like; SesterTPS, sesterterpene synthase.

The most prevalent and diverse terpenes synthesized by angiosperm TPSs are monoterpenes and sesquiterpenes. Some terpenes can be synthesized by multiple functionally characterized TPSs, while others are specific catalytic products of particular TPS genes or gene classes. This indicates that certain TPSs in angiosperms may exhibit functional redundancy, whereas others may have evolved specialized functions. The diversity of terpenes produced by TPSs in different branches may depend on the intrinsic property of TPSs, their subcellular localization, and the availability of endogenous substrates. Interestingly, many TPSs exhibit multiple substrate activities *in vitro*, yet they also display preferences for certain substrates, which may be associated with their long-term adaptive evolution within a specific subcellular compartment. Another significant finding is the functional divergence of TPSs during evolution, which significantly contributes to the vast structural diversity of terpenes in angiosperms (Fig. 6). In contrast to other TPS-a subclades, TPSs of the a1.2 subclade are capable of producing diterpenes and sesterterpenes. Members of the b1.3 subclade TPSs exhibit a greater diversity of terpene products compared to the other TPS-b subclades. Similarly, TPSs of the g1 clade produce a wider array of terpene products than those within the g2 clade. Furthermore, terpenes produced by the diverse TPSs in various organs fulfill distinct biological roles, underscoring the complexity and specificity of terpene synthesis and function in angiosperms.

With the extensive publication of angiosperm genome sequences, there is a burgeoning opportunity for a more comprehensive functional characterization of TPSs in more angiosperm species. Further exploration and improvement are necessary based on the functional characterization of more TPSs to study the expression pattern and functional divergence of the TPS gene family throughout the evolutionary process. The biosynthesis of terpenes involves not only TPSs but also other enzymes in the MEP and MVA pathways, as well as certain terpene-modifying enzymes. Future research will focus on the exploration of novel enzymes, substrates, and synthetic pathways in terpene biosynthesis, which will aid in a more thorough analysis of the extensive chemical diversity of terpenes in angiosperms. Terpenes perform a variety of biological functions, and thus, their diversity may yield fresh insights into the diversity and adaptability of angiosperms. In addition, two well-established systems (*Escherichia coli* and *Nicotiana benthamiana*) have been widely used for functional characterization of TPS genes. However, the entire field of terpene biosynthesis research, particularly species-specific terpenes, faces the immense challenge of proving the physiological significance of these compounds. The reasons for this are twofold: one is that the mechanisms by which terpenes exert physiological functions within plants are complex and may involve other genes and compounds. The other reason is primarily technical, limited by the establishment of genetic transformation methods. Apart from some model plants and crop species, transgenic technology for the majority of angiosperms, especially woody plants, is still in its infancy, representing a problem that urgently needs to be addressed in the future.

## Materials and methods

### Search and filter for functionally characterized TPSs

Data for the 222 functionally characterized TPSs in 24 angiosperms (including 15 eudicots, and 9 monocots; Supplementary Data  Tables S1) were sourced from 67 relevant publications indexed in the Web of Science. These TPSs have been experimentally validated and their substrates are well-defined (Supplementary Data  Tables S2–S6). In addition, the gene names used in this study remain consistent with those in the original literature, with the exception of a few species abbreviations that were rewritten to avoid conflicts, as detailed in Supplementary Data  Tables S1.

### Collection of functionally characterized TPS sequences

Protein sequences of functionally characterized TPSs in *A. thaliana*, *S. lycopersicum*, *V. vinifera*, and *O. sativa* were downloaded from Phytozome v13 (https://phytozome-next.jgi.doe.gov/). TPS protein sequences of *Camellia sinensis*, *C. sativus*, and *R. ovatum* were obtained from the Tea Plant Genome Database Version 1.0 (TeaPGDB v1.0, https://eplant.njau.edu.cn/tea/index.html), CuGenDBv2 (http://cucurbitgenomics.org/), and the Rhododendron Plant Genome Database (RPGD, http://bioinfor.kib.ac.cn/RPGD/), respectively. TPS protein sequences of *E. grandis*, *C. florida*, *F. hybrida*, *W. villosa*, *and Lilium* ‘Siberia’ were obtained from previous studies [43, 59, 62–64, 98]. TPS protein sequences of the remaining 11 angiosperms (*C. sativa*, *B. oleracea*, *B. rapa*, *C. rubella*, *A. julibrissin*, *Freesia caryophyllacea*, *F. viridis*, *F. corymbose*, *F. refracta*, *P. bellina*, *A. oxysepala*, *and A. japonica*) were available from the National Center for Biotechnology Information (NCBI, https://www.ncbi.nlm.nih.gov/).

### Multiple sequence alignment and phylogenetic analysis

Multisequence alignments of TPS protein sequences were generated using the MUSCLE (v5.1) program [99] with default parameters and then trimmed by TrimAl (1.4.1) [100]. The maximum-likelihood (ML) tree was constructed using IQTREE (v1.6.12) [101] with the bootstrap values performed with 1000 replicates and was visualized and beautified by MEGA (v 7.0.26) [102] and iTOL (https://itol.embl.de/) [103]. Bootstrap values less than 80 are not displayed in the tree.

### Expression analysis of functionally characterized TPSs

Expression data pertaining to the TPSs of tomato was gleaned from the RT-qPCR findings [24]. Expression data of Arabidopsis was extracted from previous research [51] and the RT-qPCR outcomes [36]. Expression data of *W. villosa* was amassed from the transcriptome and RT-qPCR results [59].

## Supplementary Material

Web_Material_uhae272

## Data Availability

No additional data were generated in this article.
