# Supplementary material for: Expansion and functional divergence of terpene synthase genes in angiosperms: a driving force of terpene diversity
Source: Hortic Res. 2024 Sep 25;12(1):uhae272. doi: 10.1093/hr/uhae272 (PMC11725647; doi:10.1093/hr/uhae272)
Supplement: Web_Material_uhae272 [file web_material_uhae272.docx]

**Supplementary Data Table S1.** A list of 24 angiosperms providing protein sequences of functionally characterized TPSs.

| **Eudicots** | **Species** | **TPS abbreviation** | **Monocots** | **Species** | **TPS abbreviation** |
| --- | --- | --- | --- | --- | --- |
|  | *Aquilegia oxysepala* | *AoTPS* |  | *Freesia x hybrida* | *FhTPS* |
|  | *Arabidopsis thaliana* | *AtTPS* |  | *Freesia viridis* | *FviTPS* |
|  | *Albizia julibrissin* | *AjTPS* |  | *Freesia refracta* | *FreTPS* |
|  | *Aquilegia japonica* | *AjaTPS* (*AjTPS^a^*) |  | *Freesia caryophyllacea* | *FcaTPS* |
|  | *Brassica oleracea* | *Bo* |  | *Freesia corymbose* | *FcoTPS* |
|  | *Brassica rapa* | *Br* |  | *Lilium* ‘Siberia’ | *LoTPS* |
|  | *Capsella rubella* | *Cr* |  | *Oryza sativa* | *OsTPS* |
|  | *Cannabis sativa* | *CsaTPS* (*CsTPS^a^*) |  | *Phalaenopsis bellina* | *PbTPS* |
|  | *Camellia sinensis* | *CsiTPS* (*CsTPS^a^*) |  | *Wurfbainia villosa* | *WvTPS* |
|  | *Cucumis sativus* | *CusTPS* (*CsTPS^a^*) |  |  |  |
|  | *Clematis florida* | *CfTPS* |  |  |  |
|  | *Eucalyptus grandis* | *EgranTPS* |  |  |  |
|  | *Rhododendron ovatum* | *RoTPS* |  |  |  |
|  | *Solanum lycopersicum* | *SlyTPS* |  |  |  |
|  | *Vitis vinifera* | *VvTPS* |  |  |  |

*^a^* The original TPS abbreviations in the references. Their designations were changed to better distinguish them in this study.

**Supplementary Data Table S2.** A list of TPS-a genes in this article, their characteristics, and the major terpenes they produce.

| **Gene ID** | **TPS name** | **Enzyme name** | **Species** | **TPS-clade** | **Subcellular localization** | **Substrate** | **Product class** | **Major terpene product** | **Reference** |
| --- | --- | --- | --- | --- | --- | --- | --- | --- | --- |
| Solyc01g101170 | SlyTPS31 | Viridiflorene synthase | *Solanum lycopersicum* | a1.2 | Cytosol | *E,E*-FPP | C15, sesquiterpene | Viridiflorene | [1, 2] |
| Solyc01g101180 | SlyTPS32 | Viridiflorene synthase | *Solanum lycopersicum* | a1.2 | Cytosol | *E,E*-FPP  *Z,Z*-FPP | C15, sesquiterpene | Viridiflorene  Unidentified | [2, 3] |
| Solyc01g101190 | SlyTPS33 |  | *Solanum lycopersicum* | a1.2 | Cytosol | *E,E*-FPP  *Z,Z*-FPP | C15, sesquiterpene | Guaia-1(10),11-diene  *β*-Acoradiene | [2] |
| Solyc01g101210 | SlyTPS35 |  | *Solanum lycopersicum* | a1.2 | Cytosol | *E,E*-FPP  *Z,Z*-FPP | C15, sesquiterpene | Guaia-1(10),11-diene  (*Z,Z*)-Farnesol |  |
| Solyc04g051620 | SlyTPS48 | Hedycaryol synthase | *Solanum lycopersicum* | a1.2 | Cytosol | *E,E*-FPP  *Z,Z*-FPP | C15, sesquiterpene | Hedycaryol  Unidentified |  |
| Solyc04g054380 | SlyTPS28 | Hedycaryol synthase | *Solanum lycopersicum* | a1.2 | Cytosol | *E,E*-FPP  *Z,Z*-FPP | C15, sesquiterpene | Hedycaryol  Unidentified |  |
| Solyc06g059885 | SlyTPS9 | Germacrene C synthase | *Solanum lycopersicum* | a1.3 | Cytosol | GPP  *E,E*-FPP  *Z,Z*-FPP | C10, monoterpene  C15, sesquiterpene | *β*-Myrcene  Germacrene C  Germacrene C | [2, 4] |
| Solyc06g059910 | SlyTPS10 | *α*-Bisabolol synthase | *Solanum lycopersicum* | a1.3 | Cytosol | *Z,Z*-FPP | C15, sesquiterpene | *α*-Bisabolol | [2] |
| Solyc06g059930 | SlyTPS12 | *β*-Caryophyllene/*α*-Humulene synthase | *Solanum lycopersicum* | a1.3 | Cytosol | GPP  *E,E*-FPP  *Z*,*Z*-FPP | C10, monoterpene  C15, sesquiterpene | *β*-Myrcene  *β*-Caryophyllene, *α*-humulene  *γ*-Curcumene, *β*-Bisabolene | [2, 5] |
| Solyc06g060180 | SlyTPS36 | *cis*-Muurola-3,5-diene synthase | *Solanum lycopersicum* | a1.3 | Mitochondria | *Z,Z*-FPP | C15, sesquiterpene | *cis*-Muurola-3,5-diene | [2, 3] |
| Solyc07g008690 | SlyTPS16 | *δ*-Cadinene synthase | *Solanum lycopersicum* | a1.3 | Cytosol | *E,E*-FPP | C15, sesquiterpene | *δ*-Cadinene | [2] |
| Solyc07g052120 | SlyTPS51 |  | *Solanum lycopersicum* | a1.3 | Cytosol | *E,E*-FPP  *Z,Z*-FPP | C15, sesquiterpene | (*E*)-Nerolidol  *α*-Bisabolol |  |
| Solyc07g052140 | SlyTPS52 |  | *Solanum lycopersicum* | a1.3 | Cytosol | *E,E*-FPP  *Z,Z*-FPP | C15, sesquiterpene | (*E*)-Nerolidol  *α*-Bisabolol |  |
| Solyc09g092470 | SlyTPS14 |  | *Solanum lycopersicum* | a1.3 | Cytosol | *E,E*-FPP  *Z,Z*-FPP | C15, sesquiterpene | *β*-Bisabolene  *α*-Bisabolene | [3] |
| Solyc12g006570 | SlyTPS17 | Valencene synthase | *Solanum lycopersicum* | a1.3 | Cytosol | GPP  *E,E*-FPP  *Z,Z*-FPP | C10, monoterpene  C15, sesquiterpene | *β*-Myrcene  Valencene  (*Z*)-*γ*-Bisabolene | [1, 2] |
| At1g33750 | AtTPS22 |  | *Arabidopsis thaliana* | a1.2 |  | *E,E*-FPP | C15, sesquiterpene | (*E*)-*β*-Farnesene, *α*-Farnesene | [6] |
| At1g66020 | AtTPS26 |  | *Arabidopsis thaliana* | a1.2 |  | GGPP | C20,diterpene | Unidentified |  |
| At1g70080 | AtTPS6 |  | *Arabidopsis thaliana* | a1.2 |  | GGPP  GFPP | C20,diterpene | Dolabelladienol  (*2E*)-Flocerene | [6, 7] |
| At3g14490 | AtTPS17 | (-)-Arathanadiene A/(-)-Arathanadiene B synthase | *Arabidopsis thaliana* | a1.2 |  | GFPP | C25,sesterterpene | (-)-Arathanadiene A, (-)-Arathanadiene B | [8] |
| At3g14520 | AtTPS18 | (+)-Thalianatriene synthase | *Arabidopsis thaliana* | a1.2 | Plastid | GFPP | C25,sesterterpene | (+)-Thalianatriene | [9, 10] |
| At3g14540 | AtTPS19 | (−)-Retigeranin B synthase | *Arabidopsis thaliana* | a1.2 | Plastid | GFPP | C25,sesterterpene | (−)-Retigeranin B | [9, 10] |
| At3g29410 | AtTPS25 | (-)-*Ent*-quiannulatene synthase | *Arabidopsis thaliana* | a1.2 | Plastid | GFPP | C25,sesterterpene | (-)-*Ent*-quiannulatene, (-)-Variculatriene A | [10, 11] |
| At3g32030 | AtTPS30 | (+)-Astallatene synthase | *Arabidopsis thaliana* | a1.2 | Plastid | GFPP | C25,sesterterpene | (+)-Astallatene | [10, 11] |
| At4g13280 | AtTPS12 | (*Z*)-*γ*-Bisabolene synthase | *Arabidopsis thaliana* | a1.2 |  | *E,E*-FPP | C15, sesquiterpene | (*Z*)-*γ*-Bisabolene | [12] |
| At4g13300 | AtTPS13 | (*Z*)-*γ*-Bisabolene synthase | *Arabidopsis thaliana* | a1.2 |  | *E,E*-FPP | C15, sesquiterpene | (*Z*)-*γ*-Bisabolene |  |
| At4g20210 | AtTPS8 | Rhizathalene synthase | *Arabidopsis thaliana* | a1.2 | Plastid | GGPP | C20,diterpene | Rhizathalene A | [13] |
| At4g20230 | AtTPS9 |  | *Arabidopsis thaliana* | a1.2 |  | GGPP | C20,diterpene | Unidentified | [6] |
| At5g23960 | AtTPS21 | (*E*)-*β*-Caryophyllene synthase | *Arabidopsis thaliana* | a1.2 |  | *E,E*-FPP | C15, sesquiterpene | (*E*)-*β*-Caryophyllene | [14] |
| At5g44630 | AtTPS11 |  | *Arabidopsis thaliana* | a1.2 |  | *E,E*-FPP | C15, sesquiterpene | (+)-*α*-Barbatene, (+)-Thujopsene | [15] |
| At5g48110 | AtTPS20 |  | *Arabidopsis thaliana* | a1.2 | Plastid | GGPP | C20,diterpene | Dolathaliatriene, Dolabelladienol | [6] |
| At1g31950 | AtTPS29 |  | *Arabidopsis thaliana* | a1.2 |  | GFPP | C25,sesterterpene | Unidentified | [7] |
| LOC106343250 | Bo250 | (+)-Boleracene synthase | *Brassica oleracea* | a1.2 |  | GFPP | C25,sesterterpene | (+)-Boleracene | [11] |
| LOC103859580 | Br580 | (+)-Brarapadiene A/(-)-Brarapadiene B synthase | *Brassica rapa* | a1.2 |  | GFPP | C25,sesterterpene | (+)-Brarapadiene A, (-)-Brarapadiene B | [8] |
| CARUB_v10016089mg | Cr089 | (+)-Caprutriene C/(-)-Caprudiene A synthase | *Capsella rubella* | a1.2 |  | GFPP | C25,sesterterpene | (+)-Caprutriene C, (-)-Caprudiene A | [8] |
| CARUB_v10016237mg | Cr237 | (−)-Caprutriene A synthase | *Capsella rubella* | a1.2 |  | GFPP | C25,sesterterpene | (−)-Caprutriene A | [11] |
| GSVIVT01014566001 | VvTPS27 | (*E*)-*β*-Caryophyllene synthase | *Vitis vinifera* | a1.1 |  | FPP | C15, sesquiterpene | (*E*)-Caryophyllene, *α*-Humulene | [16] |
| GSVIVT01036312001 | VvTPS02 | (*E*)-*β*-Caryophyllene synthase | *Vitis vinifera* | a1.1 |  | FPP | C15, sesquiterpene | (*E*)-Caryophyllene, *α*-Humulene |  |
| GSVIVT01036360001 | VvTPS13 | (*E*)-*β*-Caryophyllene synthase | *Vitis vinifera* | a1.1 |  | FPP | C15, sesquiterpene | (*E*)-Caryophyllene, *α*-Humulene |  |
| GSVIVT01036308001 | VvTPS01 | Germacrene A synthase | *Vitis vinifera* | a1.1 |  | FPP | C15, sesquiterpene | Germacrene A, *α*-Selinene |  |
| GSVIVT01036344001 | VvTPS10 | (*E*)-*α*-Bergamotene synthase | *Vitis vinifera* | a1.1 |  | FPP | C15, sesquiterpene | (*E*)-*α*-Bergamotene, Nerolidol |  |
| GSVIVT01036322001 | VvTPS07 | Germacrene D synthase | *Vitis vinifera* | a1.1 |  | FPP | C15, sesquiterpene | Germacrene D |  |
| GSVIVT01036366001 | VvTPS15 | Germacrene D synthase | *Vitis vinifera* | a1.1 |  | FPP | C15, sesquiterpene | Germacrene D |  |
| GSVIVT01014174001 | VvTPS20 | (*E,E*)-*α*-Farnesene synthase | *Vitis vinifera* | a1.1 |  | FPP | C15, sesquiterpene | (*E,E*)-*α*-Farnesene |  |
| GSVIVT01036330001 | VvTPS08 | *γ*-Cadinene synthase | *Vitis vinifera* | a1.1 |  | FPP | C15, sesquiterpene | *γ*-Cadinene |  |
| GSVIVT01036367001 | VvTPS30 | *β*-Curcumene synthase | *Vitis vinifera* | a1.1 |  | FPP | C15, sesquiterpene | *β*-Curcumene, (*E*)-*γ*-Bisabolene |  |
| GSVIVT01036351001 | VvTPS12 | Sesquithujene synthase | *Vitis vinifera* | a1.1 |  | FPP | C15, sesquiterpene | Sesquithujene |  |
| GSVIVT01036361001 | VvTPS14 | *α*-Zingiberene synthase | *Vitis vinifera* | a1.1 |  | FPP | C15, sesquiterpene | *α*-Zingiberene, *β*-Sesquiphellandrene |  |
| GSVIVT01014325001 | VvTPS24 | Selina-4,11-diene/Intermedeol synthase | *Vitis vinifera* | a1.1 |  | FPP | C15, sesquiterpene | Selina-4,11-diene, Intermedeol |  |
| GSVIVT01014558001 | VvTPS26 | Cubebol/*δ*-Cadinene synthase | *Vitis vinifera* | a1.1 |  | FPP | C15, sesquiterpene | Cubebol, *δ*-Cadinene |  |
| GSVIVT01036348001 | VvTPS11 | *α*-Humulene synthase | *Vitis vinifera* | a1.1 |  | FPP | C15, sesquiterpene | *α*-Humulene, Hyemalol |  |
| GSVIVT01014175001 | VvTPS21 | (*E*)-*β*-Caryophyllene/2-epi-(E)-*β*-Caryophyllene synthase | *Vitis vinifera* | a1.1 |  | FPP | C15, sesquiterpene | (*E*)-*β*-Caryophyllene, 2-epi-(E)-*β*-Caryophyllene |  |
| Eucgr.D01105.1 | EgranTPS013 | Isoledene/Cadinene synthase | *Eucalyptus grandis* | a1.3 |  | FPP | C15, sesquiterpene | Isoledene, Cadinene | [17] |
| Eucgr.E00419.1 | EgranTPS019 | Germacrene D synthase | *Eucalyptus grandis* | a1.3 |  | FPP | C15, sesquiterpene | Germacrene D |  |
| Eucgr.K03518.1 | EgranTPS041 | Bicyclogermacrene synthase | *Eucalyptus grandis* | a1.3 |  | FPP | C15, sesquiterpene | Bicyclogermacrene |  |
| MN654905 | AjTPS5 |  | *Albizia julibrissin* | a1.3 |  | GPP  *Z*,*Z*-FPP | C10, monoterpene  C15, sesquiterpene | (1*R*)-*α*-Pinene, *γ*-Terpinene  *α*-Farnesene, Longifolene, *α*-Himachalene | [18] |
|  | CfTPS3 |  | *Clematis florida* | a1.2 |  | *E,E*-FPP | C15, sesquiterpene | *α*-Isocomene, (*E*)-*β*-Caryophyllene,  *α*-Humulene | [19] |
|  | FhTPS6 | α-Selinene synthase | *Freesia x hybrida* | a1.4 | Cytosol | GPP  *E,E*-FPP | C10, monoterpene  C15, sesquiterpene | Myrcene, (*E*)-Ocimene, (*Z*)-Ocimene  Selinene, Nerolidol | [20] |
|  | FhTPS7 |  | *Freesia x hybrida* | a1.4 | Cytosol | GPP  *E,E*-FPP | C10, monoterpene  C15, sesquiterpene | Myrcene  Copaene |  |
|  | FhTPS8 |  | *Freesia x hybrida* | a1.4 | Cytosol | *E,E*-FPP | C15, sesquiterpene | *α*-Gurjunene |  |
|  | FviTPS10 | *β*-Caryophyllene synthase | *Freesia viridis* | a1.4 | Cytosol | *E,E*-FPP | C15, sesquiterpene | *β*-Caryophyllene | [21] |
| KY014564 | CsaTPS4 | Alloaromadendrene synthase | *Cannabis sativa* | a1.1 |  | *E,E*-FPP | C15, sesquiterpene | Alloaromadendrene | [22] |
| KY014554 | CsaTPS7 | *δ*-Selinene synthase | *Cannabis sativa* | a1.1 |  | *E,E*-FPP | C15, sesquiterpene | *δ*-Selinene |  |
| KY014556 | CsaTPS8 | *γ*-Eudesmol synthase | *Cannabis sativa* | a1.1 |  | *E,E*-FPP | C15, sesquiterpene | *γ*-Eudesmol |  |
| KY014555 | CsaTPS9 | *β*-Caryophyllene/*α*-Humulene synthase | *Cannabis sativa* | a1.1 |  | *E,E*-FPP | C15, sesquiterpene | *β*-Caryophyllene, *α*-Humulene |  |
| MK131289 | CsaTPS16 | Germacrene B synthase | *Cannabis sativa* | a1.1 |  | *E,E*-FPP | C15, sesquiterpene | Germacrene B | [23] |
| MK801762 | CsaTPS20 | Hedycaryol synthase | *Cannabis sativa* | a1.1 |  | *E,E*-FPP | C15, sesquiterpene | Hedycaryol |  |
| MN967483 | CsaTPS21 | Hedycaryol synthase | *Cannabis sativa* | a1.1 |  | *E,E*-FPP | C15, sesquiterpene | Hedycaryol | [24] |
| MN967477 | CsaTPS22 | Himachalane synthase | *Cannabis sativa* | a1.1 |  | *E,E*-FPP | C15, sesquiterpene | Himachalane |  |
| MN967472 | CsaTPS25 | (*E*)-*β*-Farnesene synthase | *Cannabis sativa* | a1.1 |  | *E,E*-FPP | C15, sesquiterpene | (*E*)-*β*-Farnesene |  |
| MN967482 | CsaTPS28 |  | *Cannabis sativa* | a1.1 |  | *E,E*-FPP | C15, sesquiterpene | *β*-Elemene |  |
| Csa2G427840 | CusTPS12 |  | *Cucumis sativus* | a1.1 |  | GPP  FPP | C10, monoterpene  C15, sesquiterpene | Linalool, Myrcene  (*E*)-*β*-Farnesene | [25] |
| Csa3G039850 | CusTPS14 |  | *Cucumis sativus* | a1.1 |  | GPP  FPP | C10, monoterpene  C15, sesquiterpene | Myrcene, Linalool  (*E*)-*β*-Farnesene |  |
| Csa3G040850 | CusTPS15 |  | *Cucumis sativus* | a1.1 |  | GPP  FPP | C10, monoterpene  C15, sesquiterpene | Linalool, (*E*)-*β*-Ocimene  (*E*)-Nerolidol, (*E*)-*β*-Farnesene |  |
| Csa3G041370 | CusTPS17 |  | *Cucumis sativus* | a1.1 |  | GPP  FPP | C10, monoterpene  C15, sesquiterpene | Linalool, Myrcene  (*E*)-*β*-Farnesene, (*E*)-Nerolidol |  |
| Csa3G095040 | CusTPS19 |  | *Cucumis sativus* | a1.1 |  | GPP  FPP  GGPP | C10, monoterpene  C15, sesquiterpene  C20,diterpene | Myrcene, Linalool  (*E,E*)-*α*-Farnesene  Geranyl linalool |  |
| Csa3G097040 | CusTPS21 |  | *Cucumis sativus* | a1.1 |  | GPP  FPP | C10, monoterpene  C15, sesquiterpene | Myrcene, Linalool  (*E*)-Caryophyllene, *α*-Humulene |  |
| Csa3G097540 | CusTPS22 |  | *Cucumis sativus* | a1.1 |  | GPP  FPP | C10, monoterpene  C15, sesquiterpene | Linalool, *α*-Terpineol, Myrcene  (*E*)-Nerolidol, Cadinol, Cadinene |  |
|  | WvTPS3 |  | *Wurfbainia villosa* | a1.4 |  | GPP  FPP | C10, monoterpene  C15, sesquiterpene | Geraniol  Humulene, Caryophyllene | [26] |
|  | WvTPS19 | *β*-Elemene synthase | *Wurfbainia villosa* | a1.4 |  | FPP | C15, sesquiterpene | *β*-Elemene |  |
|  | WvTPS25 |  | *Wurfbainia villosa* | a1.4 |  | GPP  FPP | C10, monoterpene  C15, sesquiterpene | Geraniol  Bicyclogermacrene, *γ*-Elemene |  |
|  | WvTPS26 |  | *Wurfbainia villosa* | a1.4 |  | GPP  FPP | C10, monoterpene  C15, sesquiterpene | Geraniol  Copaene, D-Germacren-4-ol |  |
|  | WvTPS27 |  | *Wurfbainia villosa* | a1.4 |  | GPP  FPP | C10, monoterpene  C15, sesquiterpene | Linalool  (*Z*)-*α*-Bergamotene, *α*-Santalene |  |
|  | WvTPS42 | *γ*-Elemene/*β*-Elemene synthase | *Wurfbainia villosa* | a1.4 |  | FPP | C15, sesquiterpene | *γ*-Elemene, *β*-Elemene |  |
|  | WvTPS58 | Aristolochene synthase | *Wurfbainia villosa* | a1.4 |  | FPP | C15, sesquiterpene | Aristolochene |  |
| LOC_Os01g23530.1 | OsTPS1 | *α*-Bisabolol synthase | *Oryza sativa* | a1.4 | Cytosol | FPP | C15, sesquiterpene | *α*-Bisabolol | [27] |
| Os08g07100 | Os100 |  | *Oryza sativa* | a1.4 |  | FPP | C15, sesquiterpene | Zingiberene, *β*-Sesquiphellandrene，*β*-Bisabolene，(*E*)-*γ*-Bisabolene | [28, 29] |
| Os08g07120 | Os120 |  | *Oryza sativa* | a1.4 |  | FPP | C15, sesquiterpene | *β*-Bisabolene，(*E*)-*β*-Farnesene | [29] |
| LOC_Os04g27430 | OsTPS2 | (*E*)-*β*-Farnesene synthase | *Oryza sativa* | a1.4 |  | - | - | (*E*)-*β*-Farnesene (in vivo) | [30] |
| Os08g04500 | OsTPS3 | (*E*)-*β*-Caryophyllene synthase | *Oryza sativa* | a1.4 |  | FPP | C15, sesquiterpene | (*E*)-*β*-Caryophyllene, *β*-Elemene | [31] |
| LOC_Os07g11790.1 | OsTPS13 | (*E,E*)-Farnesol synthase | *Oryza sativa* | a1.4 |  | FPP | C15, sesquiterpene | (*E,E*)-Farnesol, Nerolidol |  |
| Os04g27070 | OsTPS18 | (*E*)-Nerolidol/(*E*)-*β*-Farnesene synthase | *Oryza sativa* | a1.4 | Cytosol | FPP | C15, sesquiterpene | (*E*)-Nerolidol，(*E*)-*β*-Farnesene | [32] |
| Os04g27190 | OsTPS19 | (*S*)-Limonene synthase | *Oryza sativa* | a1.4 | plastids | GPP  FPP | C10, monoterpene  C15, sesquiterpene | (*S*)-Limonene  *β*-Bisabolene, *β*-Elemene | [33] |
| Os04g27340 | OsTPS20 | (*S*)-Limonene synthase | *Oryza sativa* | a1.4 | plastids | GPP  FPP | C10, monoterpene  C15, sesquiterpene | (*S*)-Limonene, *cis*-Sabinene, *γ*-Terpinene  *β*-Bisabolene, Zingiberene |  |
| XP_015635171 | OsTPS21 | Geraniol synthase | *Oryza sativa* | a1.4 | chloroplasts | GPP | C10, monoterpene | Geraniol | [34] |
| Os04g27790 | OsTPS24 | *γ*-Terpinene synthase | *Oryza sativa* | a1.4 | chloroplasts | GPP  FPP | C10, monoterpene  C15, sesquiterpene | *γ*-Terpinene  Unidentified | [35] |

**Supplementary Data Table S3.** A list of TPS-b genes in this article, their characteristics, and the major terpenes they produce.

| **Gene ID** | **TPS name** | **Enzyme name** | **Species** | **TPS-clade** | **Subcellular localization** | **Substrate** | **Product class** | **Major terpene product** | **Reference** |
| --- | --- | --- | --- | --- | --- | --- | --- | --- | --- |
| Solyc01g105870 | SlyTPS3 | Camphene synthase | *Solanum lycopersicum* | b1.1 | Plastid | GPP | C10, monoterpene | Camphene, Tricyclene | [2, 3] |
| Solyc01g105880 | SlyTPS4 | *β*-Phellandrene synthase | *Solanum lycopersicum* | b1.1 | Plastid | GPP | C10, monoterpene | *β*-Phellandrene | [2, 36] |
| Solyc01g105890 | SlyTPS5 | Linalool synthase | *Solanum lycopersicum* | b1.1 | Plastid | GPP  *E,E*-FPP | C10, monoterpene  C15, sesquiterpene | Linalool  (*E*)-Nerolidol | [2, 36] |
| Solyc01g105920 | SlyTPS7 |  | *Solanum lycopersicum* | b1.1 | Plastid | GPP | C10, monoterpene | *β*-Myrcene, Limonene | [2, 3] |
| Solyc01g105960 | SlyTPS8 | 1,8-Cineole synthase | *Solanum lycopersicum* | b1.1 | Cytosol | GPP  NPP | C10, monoterpene C10, monoterpene | 1,8-Cineole  1,8-Cineole | [2, 3] |
| Solyc02g079840 | SlyTPS38 | *α*-Bergamotene synthase | *Solanum lycopersicum* | b1.1 | Cytosol | E,E-FPP | C15, sesquiterpene | *α*-Bergamotene | [2, 3] |
| Solyc02g079890 | SlyTPS25 | *β*-Ocimene synthase | *Solanum lycopersicum* | b1.3 | Cytosol | GPP | C10, monoterpene | *β*-Ocimene | [2] |
| Solyc02g079910 | SlyTPS27 | α-Farnesene synthase | *Solanum lycopersicum* | b1.3 | Cytosol | E,E-FPP | C15, sesquiterpene | *α*-Farnesene |  |
| Solyc03g007730 | SlyTPS47 | Isoprene synthase | *Solanum lycopersicum* | b1.3 | Cytosol | DMAPP | C5, hemiterpene | Isoprene |  |
| At2g24210 | AtTPS10 | (*E*)-*β*-Ocimene/Myrcene synthase | *Arabidopsis thaliana* | b1.1 | Plastid | GPP | C10, monoterpene | (*E*)-*β*-Ocimene, Myrcene | [37] |
| At3g25810 | AtTPS24 |  | *Arabidopsis thaliana* | b1.1 |  | GPP | C10, monoterpene | (*E*)-*β*-Ocimene, *β*-Myrcene, *α*-Pinene, Sabinene, *β*-Pinene, Limonene | [14] |
| At3g25820 | AtTPS27 | 1,8-Cineole synthase | *Arabidopsis thaliana* | b1.1 | Plastid | GPP | C10, monoterpene | 1,8-Cineole | [38, 39] |
| At3g25830 | AtTPS23 | 1,8-Cineole synthase | *Arabidopsis thaliana* | b1.1 | Plastid | GPP | C10, monoterpene | 1,8-Cineole | [38] |
| At4g16730 | AtTPS2 | (*E*)-*β*-Ocimene synthase | *Arabidopsis thaliana* | b1.1 | Plastid | GPP  *E,E*-FPP | C10, monoterpene  C15, sesquiterpene | (*E*)-*β*-Ocimene, Myrcene  (*E,E*)-*α*-Farnesene | [40] |
| At4g16740 | AtTPS3 | (*E*)-*β*-Ocimene synthase | *Arabidopsis thaliana* | b1.1 | Plastid | GPP  *E,E*-FPP | C10, monoterpene  C15, sesquiterpene | (*E*)-*β*-Ocimene, Myrcene  (*E,E*)-*α*-Farnesene | [41] |
| HM807382 | VvTPS45 | (+)-*α*-Phellandrene synthase | *Vitis vinifera* | b1.1 |  | GPP | C10, monoterpene | (+)-*α*-Phellandrene, Myrcene, Terpinolene | [16] |
| GSVIVT01013518001 | VvTPS44 | (+)-*α*-Pinene synthase | *Vitis vinifera* | b1.1 |  | GPP | C10, monoterpene | (+)-*α*-Pinene, (+)-Limonene |  |
| GSVIVT01000401001 | VvTPS34 | (*E*)-*β*-Ocimene synthase | *Vitis vinifera* | b1.2 |  | GPP | C10, monoterpene | (*E*)-*β*-Ocimene |  |
| GSVIVT01000402001 | VvTPS35 | (*E*)-*β*-Ocimene synthase | *Vitis vinifera* | b1.2 |  | GPP | C10, monoterpene | (*E*)-*β*-Ocimene |  |
| HM807387 | VvTPS38 | (*E*)-*β*-Ocimene/Myrcene synthase | *Vitis vinifera* | b1.1 |  | GPP | C10, monoterpene | (*E*)-*β*-Ocimene, Myrcene |  |
| GSVIVT01006642001 | VvTPS47 | (*E*)-*β*-Ocimene/(*E,E*)-*α*-Farnesene synthase | *Vitis vinifera* | b1.3 |  | GPP  FPP | C10, monoterpene  C15, sesquiterpene | (*E*)-*β*-Ocimene  (*E,E*)-*α*-Farnesene |  |
| GSVIVT01033458001 | VvTPS31 | (*3R*)-Linalool synthase | *Vitis vinifera* | b2 |  | GPP | C10, monoterpene | (*3R*)-Linalool |  |
|  | EgranTPS059 | *γ*-Terpinene synthase | *Eucalyptus grandis* | b2 |  | GPP | C10, monoterpene | *γ*-Terpinene | [17] |
| MN654902 | AjTPS2 |  | *Albizia julibrissin* | b1.3 |  | *E,E*-FPP | C15, sesquiterpene | *α*-Farnesene | [18] |
| MN654907 | AjTPS7 |  | *Albizia julibrissin* | b1.2 |  | GPP | C10, monoterpene | *β*-Ocimene |  |
| MN654909 | AjTPS9 |  | *Albizia julibrissin* | b1.1 |  | GPP | C10, monoterpene | 1R-*α*-Pinene,*γ*-Terpinene |  |
| MN654910 | AjTPS10 |  | *Albizia julibrissin* | b1.1 |  | GPP | C10, monoterpene | Linalool |  |
|  | CfTPS2 |  | *Clematis florida* | b2 |  | GPP  *E,E*-FPP | C10, monoterpene  C15, sesquiterpene | Linalool  Nerolidol | [19] |
|  | FhTPS1 | Linalool synthase | *Freesia x hybrida* | b1.4 | Plastid | GPP | C10, monoterpene | Linalool | [20] |
|  | FhTPS2 | *α*-Terpineol synthase | *Freesia x hybrida* | b1.4 | Plastid | GPP | C10, monoterpene | *α*-Terpineol |  |
| ON777840 | FviTPS12 |  | *Freesia viridis* | b1.4 | Plastid | GPP | C10, monoterpene | *α*-Thujene, Abinene, *γ*-Terpinene | [21] |
| ON777841 | FcoTPS13 |  | *Freesia corymbose* | b1.4 | Cytosol | GPP  NPP  *E,E*-FPP  *Z*,*Z*-FPP | C10, monoterpene  C10, monoterpene  C15, sesquiterpene  C15, sesquiterpene | (1*R*)-(+)-*α*-Pinene, (1*S*)-(−)-*β*-Pinene,  (+)-Limonene  Sabinene,(+)-Limonene  (E)-*β*-Farnesene  (E)-*β*-Farnesene |  |
| ON777843 | FreTPS15 |  | *Freesia refracta* | b1.4 | Plastid | GPP  NPP  *E,E*-FPP  *Z*,*Z*-FPP | C10, monoterpene  C10, monoterpene  C15, sesquiterpene  C15, sesquiterpene | Sabinene  *α*-Terpineol  *α*-Bergamotene, *β*-Bisabolene  *α*-Bergamotene |  |
|  | LoTPS4 |  | *Lilium* ‘Siberia’ | b1.4 | Plastid | GPP  FPP | C10, monoterpene  C15, sesquiterpene | (+)-Limonene, *β*-Myrcene  (*E*)-*α*-Bergamotene | [42] |
|  | LoTPS1 | (*Z*)-*β*-Ocimene/(±)-Linalool synthase | *Lilium* ‘Siberia’ | b2 | Plastid | GPP | C10, monoterpene | (*Z*)-*β*-Ocimene , (±)-Linalool | [43] |
| MW645242 | PbTPS5 | Linalool/Geraniol synthase | *Phalaenopsis bellina* | b1.4 |  | GDP | C10, monoterpene | Linalool, Geraniol | [44] |
| MW645244 | PbTPS9 | Geraniol synthase | *Phalaenopsis bellina* | b1.4 |  | GDP | C10, monoterpene | Geraniol |  |
| MW645245 | PbTPS10 | Linalool synthase | *Phalaenopsis bellina* | b1.4 |  | GDP | C10, monoterpene | Linalool |  |
| ABI21837.1 | CsaTPS1 | (-)-Limonene synthase | *Cannabis sativa* | b1.1 |  | GPP | C10, monoterpene | (-)-Limonene | [45] |
| ABI21838.1 | CsaTPS2 | (+)-*α*-Pinene synthase | *Cannabis sativa* | b1.1 |  | GPP | C10, monoterpene | (+)-*α*-Pinene |  |
| KY014561 | CsaTPS3 | *β*-Myrcene synthase | *Cannabis sativa* | b1.1 |  | GPP | C10, monoterpene | Myrcene | [22] |
| MN967481 | CsaTPS5 | *β*-Myrcene synthase | *Cannabis sativa* | b1.1 |  | GPP  *E,E*-FPP | C10, monoterpene  C15, sesquiterpene | Myrcene  Bisabolol | [24] |
| KY014563 | CsaTPS6 | (*E*)-*β*-Ocimene synthase | *Cannabis sativa* | b1.1 |  | GPP | C10, monoterpene | (*E*)-*β*-Ocimene | [22] |
| KY014558 | CsaTPS13 | (*Z*)-*β*-ocimene synthase | *Cannabis sativa* | b1.1 |  | GPP | C10, monoterpene | (*Z*)-*β*-ocimene |  |
| MK801766 | CsaTPS14 | (-)-Limonene synthase | *Cannabis sativa* | b1.1 |  | GPP | C10, monoterpene | (-)-Limonene | [23] |
| MK801765 | CsaTPS15 | *β*-Myrcene synthase | *Cannabis sativa* | b1.1 |  | GPP | C10, monoterpene | Myrcene |  |
| MN967470 | CsaTPS17 | Myrcene synthase | *Cannabis sativa* | b1.1 |  | GPP | C10, monoterpene | Myrcene | [24] |
| MN967480 | CsaTPS23 | Myrcene synthase | *Cannabis sativa* | b1.1 |  | GPP | C10, monoterpene | Myrcene |  |
| MN967474 | CsaTPS31 | Terpinolene synthase | *Cannabis sativa* | b1.1 |  | GPP | C10, monoterpene | Terpinolene |  |
| MN967484 | CsaTPS32 |  | *Cannabis sativa* | b1.1 |  | GPP  *E,E*-FPP | C10, monoterpene  C15, sesquiterpene | Geraniol  Himachalane |  |
| MK614216 | CsaTPS37 | Terpinolene synthase | *Cannabis sativa* | b1.1 |  | GPP | C10, monoterpene | Terpinolene | [46] |
| MK614217 | CsaTPS38 | (*E*)-*β*-Ocimene synthase | *Cannabis sativa* | b1.1 |  | GPP | C10, monoterpene | (*E*)-*β*-Ocimene |  |
| MN967468 | CsaTPS29 | Linalool synthase | *Cannabis sativa* | b2 |  | GPP | C10, monoterpene | Linalool | [24] |
| CSS0024726.1 | CsiTPS08 | Linalool synthase | *Camellia sinensis* | b2 | Chloroplast | GPP | C10, monoterpene | Linalool | [47] |
| CSS0027229.1 | CsiTPS10 | Geraniol synthase | *Camellia sinensis* | b1.2 | Chloroplast | GPP | C10, monoterpene | Geraniol |  |
| CSS0045875.1 | CsiTPS58 | *α*-Pinene/(+)-Limonene synthase | *Camellia sinensis* | b1.1 | Chloroplast | GPP | C10, monoterpene | *α*-Pinene, (+)-Limonene |  |
| Ro_38431.3 | RoTPS059 | 3-Carene synthase | *Rhododendron ovatum* | b1.2 | Plastid | GPP  FPP | C10, monoterpene  C15, sesquiterpene | 3-Carene, Linalool, (E)-*β*-Ocimene  (Z)-*β*-Farnesene, *α*-Farnesene, Nerolidol | [48] |
| Ro_42833.1 | RoTPS072 | *α*-Farnesene synthase | *Rhododendron ovatum* | b1.2 | Cytosol | GPP  FPP | C10, monoterpene  C15, sesquiterpene | Linalool, 3-Carene, *β*-Myrcene  *α*-Farnesene | [48] |
| MZ666959 | AoTPS7 | (+)-Limonene synthase | *Aquilegia oxysepala* | b1.2 | Plastid | GPP  NPP  *E,E*-FPP | C10, monoterpene  C10, monoterpene  C15, sesquiterpene | (+)-Limonene  (+)-Limonene  (+)-*α*-Longipinene, *α*-Bergamotene | [49] |
| MZ670776 | AjaTPS8 | *β*-Sesquiphelldrene synthase | *Aquilegia japonica* | b1.2 | Cytosol | GPP  NPP  *E,E*-FPP  *Z*,*Z*-FPP | C10, monoterpene  C10, monoterpene  C15, sesquiterpene  C15, sesquiterpene | (+)-Limonene  (+)-Limonene  *β*-Sesquiphelldrene, *β*-Bisabolene  *β*-Bisabolene, *β*-Sesquiphellandrene |  |
| MZ666964 | AoTPS9 | Pinene synthase | *Aquilegia oxysepala* | b2 | Plastid | GPP  NPP | C10, monoterpene | (1*S*)-(−)-*β*-Pinene, (1*R*)-(+)-*α*-Pinene  (1*S*)-(−)-*β*-Pinene |  |
| Csa2G298300 | *CusTPS4* |  | *Cucumis sativus* | b1.1 |  | GPP  FPP | C10, monoterpene  C15, sesquiterpene | Linalool  (E)-*β*-Farnesene | [25] |
| Csa2G298310 | *CusTPS5* |  | *Cucumis sativus* | b1.1 |  | GPP  FPP | C10, monoterpene  C15, sesquiterpene | (*E*)-*β*-Ocimene  (*E*)-*β*-Farnesene,(*Z;E*)-*α*-farnesene |  |
| Csa2G299870 | *CusTPS9* |  | *Cucumis sativus* | b1.1 |  | GPP  FPP | C10, monoterpene  C15, sesquiterpene | (*E*)-*β*-ocimene  (*E*)-*β*-farnesene |  |
| Csa2G299890 | *CusTPS10* |  | *Cucumis sativus* | b1.1 |  | GPP  FPP | C10, monoterpene  C15, sesquiterpene | *α*-Terpineol,Sabinene, *β*-Pinene,*α*-Pinene  (*E*)-*β*-Farnesene, (*E;E*)-*α*-Farnesene |  |
| Csa2G299920 | *CusTPS11* |  | *Cucumis sativus* | b1.1 |  | GPP  FPP | C10, monoterpene  C15, sesquiterpene | Linalool, Myrcene  (*E*)-*β*-Farnesene |  |
|  | WvTPS11 | Terpinolene synthase | *Wurfbainia villosa* | b1.4 |  | GPP | C10, monoterpene | Terpinolene | [26] |
|  | WvTPS13 | Limonene synthase | *Wurfbainia villosa* | b1.4 |  | GPP | C10, monoterpene | Limonene, *β*-Mycrene |  |
|  | WvTPS14 |  | *Wurfbainia villosa* | b1.4 |  | GPP | C10, monoterpene | Bornyl diphosphate, Camphene, Limonene |  |
|  | WvTPS35 | Linalool/Nerolidol synthase | *Wurfbainia villosa* | b1.4 |  | GPP  FPP | C10, monoterpene  C15, sesquiterpene | Linalool  Nerolidol |  |
|  | WvTPS37 | Linalool synthase | *Wurfbainia villosa* | b1.4 |  | GPP | C10, monoterpene | Linalool |  |
|  | WvTPS39 | *β*-Ocimene synthase | *Wurfbainia villosa* | b1.4 |  | GPP | C10, monoterpene | *β*-Ocimene |  |
|  | WvTPS41 | *β*-Ocimene synthase | *Wurfbainia villosa* | b1.4 |  | GPP | C10, monoterpene | *β*-Ocimene |  |
|  | WvTPS63 | *β*-Pinene/*α*-Pinene synthase | *Wurfbainia villosa* | b1.4 |  | GPP | C10, monoterpene | *β*-Pinene, *α*-Pinene |  |

**Supplementary Data Table S4.** A list of TPS-g genes in this article, their characteristics, and the major terpenes they produce.

| **Gene ID** | **TPS name** | **Enzyme name** | **Species** | **TPS-clade** | **Subcellular localization** | **Substrate** | **Product class** | **Major terpene product** | **Reference** |
| --- | --- | --- | --- | --- | --- | --- | --- | --- | --- |
| Solyc10g005390 | SlyTPS39 | Linalool/Nerolidol synthase | *Solanum lycopersicum* | g1 | Cytosol | GPP  *E,E*-FPP | C10, monoterpene  C15, sesquiterpene | Linalool  (*E*)-Nerolidol | [2, 3] |
| Solyc10g005410 | SlyTPS37 | Linalool/Nerolidol synthase | *Solanum lycopersicum* | g1 | Cytosol | GPP  *E,E*-FPP | C10, monoterpene  C15, sesquiterpene | Linalool  (*E*)-Nerolidol | [2, 3] |
| At1g61680 | AtTPS14 | Linalool synthase | *Arabidopsis thaliana* | g1 | Plastid | GPP | C10, monoterpene | Linalool | [14] |
| GSVIVT01005221001 | VvTPS54 | (*3S*)-Linalool/(*E*)-Nerolidol synthase | *Vitis vinifera* | g1 |  | GPP  FPP | C10, monoterpene  C15, sesquiterpene | (*3S*)-Linalool  (*E*)-Nerolidol | [16] |
| GSVIVT01005272001 | VvTPS56 | (*3S*)-Linalool/(*E*)-Nerolidol synthase | *Vitis vinifera* | g1 |  | GPP  FPP | C10, monoterpene  C15, sesquiterpene | (*3S*)-Linalool  (*E*)-Nerolidol |  |
| GSVIVT01006466001 | VvTPS57 | (*3S*)-Linalool/(*E*)-Nerolidol/(*E,E*)-Geranyl linalool synthase | *Vitis vinifera* | g1 |  | GPP  FPP  GGPP | C10, monoterpene  C15, sesquiterpene  C20,diterpene | (*3S*)-Linalool  (*E*)-Nerolidol  (*E,E*)-Geranyl linalool |  |
| GSVIVT01002718001 | VvTPS63 | (*3S*)-Linalool/(*E*)-Nerolidol/(*E,E*)-Geranyl linalool synthase | *Vitis vinifera* | g1 |  | GPP  FPP  GGPP | C10, monoterpene  C15, sesquiterpene  C20,diterpene | (*3S*)-Linalool  (*E*)-Nerolidol  (*E,E*)-Geranyl linalool |  |
| GSVIVT01006467001 | VvTPS58 | (*3S*)-Linalool/(*E*)-Nerolidol/(*E,E*)-Geranyl linalool synthase | *Vitis vinifera* | g1 |  | GPP  FPP  GGPP | C10, monoterpene  C15, sesquiterpene  C20,diterpene | (*3S*)-Linalool  (*E*)-Nerolidol  (*E,E*)-Geranyl linalool |  |
| HM807397 | VvTPS61 | (*3S*)-Linalool/(*E*)-Nerolidol/(*E,E*)-Geranyl linalool synthase | *Vitis vinifera* | g1 |  | GPP  FPP  GGPP | C10, monoterpene  C15, sesquiterpene  C20,diterpene | (*3S*)-Linalool  (*E*)-Nerolidol  (*E,E*)-Geranyl linalool |  |
| GSVIVT01000414001 | VvTPS52 | Geraniol synthase | *Vitis vinifera* | g2 |  | GPP | C10, monoterpene | Geraniol |  |
| Eucgr.E03562.1 | EgranTPS101 | *β*-Pinene synthase | *Eucalyptus grandis* | g1 |  | GPP | C10, monoterpene | *β*-Pinene | [17] |
|  | CfTPS1 |  | *Clematis florida* | g1 |  | GPP  *E,E*-FPP | C10, monoterpene  C15, sesquiterpene | Linalool  Nerolidol | [19] |
|  | FhTPS4 |  | *Freesia x hybrida* | g1 | Plastid | GPP  FPP | C10, monoterpene  C15, sesquiterpene | Linalool  (*E*)-Nerolidol | [20] |
| ON777837 | FcaTPS9 |  | *Freesia caryophyllacea* | g1 | Plastid | GPP  *E,E*-FPP | C10, monoterpene  C15, sesquiterpene | Linalool  (*E*)-Nerolidol | [21] |
| ON777842 | FreTPS14 | Geraniol/Nerol synthase | *Freesia refracta* | g2 | Plastid | GPP  NPP | C10, monoterpene | Geraniol  Nerol |  |
|  | LoTPS3 | (±)-Linalool/(*Z*)-Nerolidol synthase | *Lilium* ‘Siberia’ | g1 | Mitochondria | GPP  FPP | C10, monoterpene  C15, sesquiterpene | (±)-Linalool  (*Z*)-Nerolidol | [43] |
| MN967473 | CsaTPS18 | Linalool synthase | *Cannabis sativa* | g1 |  | GPP | C10, monoterpene | Linalool | [24] |
| MK801763 | CsaTPS19 | (*E*)-Nerolidol/Linalool synthase | *Cannabis sativa* | g1 |  | GPP  *E,E*-FPP | C10, monoterpene  C15, sesquiterpene | Linalool  Nerolidol | [23] |
| MN967475 | CsaTPS35 | Linalool/Nerolidol synthase | *Cannabis sativa* | g1 |  | GPP  *E,E*-FPP | C10, monoterpene  C15, sesquiterpene | Linalool  Nerolidol | [24] |
| Csa1G066550 | CusTPS1 |  | *Cucumis sativus* | g1 |  | GPP  FPP | C10, monoterpene  C15, sesquiterpene | Linalool  (*E*)-Nerolidol,(*E*)-*β*-Farnesene | [25] |
| Csa1G066560 | CusTPS2 |  | *Cucumis sativus* | g1 |  | GPP  FPP | C10, monoterpene  C15, sesquiterpene | Linalool  (*E*)-Nerolidol |  |
| Csa1G068570 | CusTPS3 |  | *Cucumis sativus* | g1 |  | GPP  FPP | C10, monoterpene  C15, sesquiterpene | Linalool  (*E*)-*β*-Farnesene |  |
|  | WvTPS6 | (*E*)-Nerolidol synthase | *Wurfbainia villosa* | g1 |  | FPP | C15, sesquiterpene | (*E*)-Nerolidol | [26] |
| Os02g02930 | OsLIS | (*3S*)-Linalool synthase | *Oryza sativa* | g1 |  | GPP | C10, monoterpene | (*3S*)-Linalool | [28, 50] |

**Supplementary Data Table S5.** A list of TPS-e/f genes in this article, their characteristics, and the major terpenes they produce.

| **Gene ID** | **TPS name** | **Enzyme name** | **Species** | **TPS-clade** | **Subcellular localization** | **Substrate** | **Product class** | **Major terpene product** | **Reference** |
| --- | --- | --- | --- | --- | --- | --- | --- | --- | --- |
| Solyc03g006550 | SlyTPS46 | Geranyllinalool synthase | *Solanum lycopersicum* | f | Cytosol | GGPP | C20,diterpene | Geranyl linalool | [2, 51] |
| Solyc07g066670 | SlyTPS24 | ent-Kaurene synthase | *Solanum lycopersicum* | e | Plastid | CPP | C20,diterpene | Ent-kaurene | [2, 3] |
| Solyc08g005640 | SlyTPS21 | Lycosantalene synthase | *Solanum lycopersicum* | e | Plastid | NNPP | C20,diterpene | Lycosantalene | [2, 52] |
| Solyc08g005665 | SlyTPS20 | Phellandrene synthase | *Solanum lycopersicum* | e | Plastid | NPP | C10, monoterpene | *β*-Phellandrene | [2, 53] |
| Solyc08g005670 | SlyTPS19 |  | *Solanum lycopersicum* | e | Plastid | NPP | C10, monoterpene | *β*-Myrcene, *β*-Ocimene | [2, 52] |
| Solyc08g005720 | SlyTPS18 |  | *Solanum lycopersicum* | e | Mitochondria | NNPP | C20,diterpene | Unidentified | [2] |
| At1g61120 | AtTPS4 | Geranyllinalool synthase | *Arabidopsis thaliana* | f | Cytosol | GGPP | C20,diterpene | Geranyl linalool | [54] |
| At1g79460 | AtTPS32 | *Ent*-kaurene synthase | *Arabidopsis thaliana* | e | Plastid | *Ent-*CPP | C20,diterpene | *Ent*-kaurene | [55, 56] |
|  | LoTPS2 | (*E,E*)-*α*-Farnesene synthase | *Lilium* ‘Siberia’ | f | Cytosol | FPP | C15, sesquiterpene | (*E,E*)-*α*-Farnesene | [42] |
| MW645240 | PbTPS3 | Linalool/(*Z*)-*β*-Ocimene synthase | *Phalaenopsis bellina* | f | Chloroplast | GPP | C10, monoterpene | Linalool, (*Z*)-*β*-Ocimene | [44] |
| MW645241 | PbTPS4 | Linalool synthase | *Phalaenopsis bellina* | f | Chloroplast | GPP | C10, monoterpene | Linalool |  |
| MT295505 | CsaTPS65 |  | *Cannabis sativa* | e |  | GGPP | C20,diterpene | Copalyl diphosphate | [24] |
| Csa7G239640 | CusTPS24 |  | *Cucumis sativus* | e |  | GPP  FPP  GGPP | C10, monoterpene  C15, sesquiterpene  C20,diterpene | (*E*)-*β*-Ocimene  Cadinol, (*E*)-Nerolidol  Geranyl linalool | [25] |
| Os04g52230 | OsKS1 | *Ent*-kaurene synthase | *Oryza sativa* | e |  | *Ent-*CPP | C20,diterpene | *Ent*-kaurene | [57] |
| Os04g52240 | OsKSL2 | *Ent-*beyerene synthase | *Oryza sativa* | e | Chloroplast | *Ent-*CPP | C20,diterpene | *Ent-*beyerene | [58] |
| Os04g10060 | OsKSL4 | *Syn-*pimara-7,15-diene synthase | *Oryza sativa* | e |  | *syn*-CPP | C20,diterpene | *Syn-*pimara-7,15-diene | [59, 60] |
| Os02g36220 | OsKSL5 | *Ent*-pimara-8(14),15-diene synthase | *Oryza sativa* | e |  | *Ent-*CPP | C20,diterpene | *Ent*-pimara-8(14),15-diene | [61] |
| Os02g36264 | OsKSL6 | *Ent*-kaur-15-ene synthase | *Oryza sativa* | e |  | *Ent-*CPP | C20,diterpene | *Ent*-kaur-15-ene |  |
| Os02g36140 | OsKSL7 | *Ent*-cassa12,15-diene synthase | *Oryza sativa* | e |  | *Ent-*CPP | C20,diterpene | *Ent*-cassa12,15-diene | [62] |
| Os11g28530 | OsKSL8 | *Syn*-stemar-13-ene synthase | *Oryza sativa* | e |  | *syn*-CPP | C20,diterpene | *Syn*-stemar-13-ene | [63] |
| Os12g30824 | OsKSL10 | *Ent*-sandaraco-pimaradiene/  *Syn*-labda-8(14),15-diene synthase | *Oryza sativa* | e |  | *Ent-*CPP  *syn*-CPP | C20,diterpene | *Ent*-sandaraco-pimaradiene  *Syn*-labda-8(14),15-diene | [60, 64] |
| Q1AHB2 | OsKSL11 | *Syn*-stemod13(17)-ene synthase | *Oryza sativa* | e |  | *syn*-CPP | C20,diterpene | *Syn*-stemod13(17)-ene | [65] |

**Supplementary Data Table S6.** A list of TPS-c genes in this article, their characteristics, and the major terpenes they produce.

| **Gene ID** | **TPS name** | **Enzyme name** | **Species** | **TPS-clade** | **Subcellular localization** | **Substrate** | **Product class** | **Major terpene product** | **Reference** |
| --- | --- | --- | --- | --- | --- | --- | --- | --- | --- |
| Solyc06g084240 | SlyTPS40 | Copalyl diphosphate synthase | *Solanum lycopersicum* | c | Plastid | GGPP | C20,diterpene | Copalyl diphosphate | [2, 66] |
| Solyc08g005710 | SlyTPS41 | Copalyl diphosphate synthase | *Solanum lycopersicum* | c | Mitochondria | GGPP | C20,diterpene | Copalyl diphosphate | [2] |
| At4g02780 | AtTPS31 | *ent*-CPP synthase | *Arabidopsis thaliana* | c | Plastid | GGPP | C20,diterpene | *Ent*-CPP | [56] |
| MT295506 | CsaTPS66 |  | *Cannabis sativa* | c |  | - | - | *Ent*-kaurene (putative product) | [24] |
| Csa6G410650 | CusTPS23 |  | *Cucumis sativus* | c |  | GPP  FPP | C10, monoterpene  C15, sesquiterpene | Myrcene, Linalool  (*E*)-*β*-farnesene,(*E*)-nerolidol | [25] |
|  | WvTPS53 | Copalyl diphosphate synthase | *Wurfbainia villosa* | c |  | GGPP | C20,diterpene | Copalyl diphosphate | [26] |
|  | WvTPS59 | Copalyl diphosphate synthase | *Wurfbainia villosa* | c |  | GGPP | C20,diterpene | Copalyl diphosphate |  |
| Q6ET36 | OsCPS1 | *ent-*CPS | *Oryza sativa* | c |  | GGPP | C20,diterpene | *ent*-CPP | [67, 68] |
| Q6Z5I0 | OsCPS2 | *ent-*CPS | *Oryza sativa* | c |  | GGPP | C20,diterpene | *ent-*CPP | [67, 68] |
| Q0JF02 | OsCPS4 | *syn*-CPS | *Oryza sativa* | c |  | GGPP | C20,diterpene | *syn*-CPP | [67] |

**References**

1. Bleeker PM, Spyropoulou EA, Diergaarde PJ. et al. RNA-seq discovery, functional characterization, and comparison of sesquiterpene synthases from *Solanum lycopersicum* and *Solanum habrochaites* trichomes. *Plant Mol Biol*. 2011;**77**:323-36.

2. Zhou F, Pichersky E. The complete functional characterisation of the terpene synthase family in tomato. *New Phytol*. 2020;**226**:1341-60.

3. Falara V, Akhtar TA, Nguyen TTH. et al. The tomato terpene synthase gene family. *Plant Physiol*. 2011;**157**:770-89.

4. Colby SM, Crock J, Dowdle-Rizzo B. et al. Germacrene C synthase from *Lycopersicon esculentum* cv. VFNT cherry tomato: cDNA isolation, characterization, and bacterial expression of the multiple product sesquiterpene cyclase. *Proc Natl Acad Sci U S A*. 1998;**95**:2216-21.

5. Schilmiller AL, Miner DP, Larson M. et al. Studies of a biochemical factory: tomato trichome deep expressed sequence tag sequencing and proteomics. *Plant Physiol*. 2010;**153**:1212-23.

6. Wang Q, Jia M, Huh JH. et al. Identification of a dolabellane type diterpene synthase and other root-expressed diterpene synthases in *Arabidopsis*. *Front Plant Sci*. 2016;**7**:1761.

7. Chen Q, Li J, Liu Z. et al. Molecular basis for sesterterpene diversity produced by plant terpene synthases. *Plant Commun*. 2020;**1**:100051.

8. Huang AC, Hong YJ, Bond AD. et al. Diverged plant terpene synthases reroute the carbocation cyclization path towards the formation of unprecedented 6/11/5 and 6/6/7/5 sesterterpene scaffolds. *Angew Chem Int Ed Engl*. 2018;**57**:1291-5.

9. Shao J, Chen QW, Lv H. et al. (+)-Thalianatriene and (−)-retigeranin B catalyzed by sesterterpene synthases from *Arabidopsis thaliana*. *Org Lett*. 2017;**19**:1816-9.

10. Chen Q, Jiang T, Liu YX. et al. Recently duplicated sesterterpene (C25) gene clusters in *Arabidopsis thaliana* modulate root microbiota. *Sci China Life Sci*. 2019;**62**:947-58.

11. Huang AC, Kautsar SA, Hong YJ. et al. Unearthing a sesterterpene biosynthetic repertoire in the Brassicaceae through genome mining reveals convergent evolution. *Proc Natl Acad Sci U S A*. 2017;**114**:E6005-14.

12. Ro DK, Ehlting J, Keeling CI. et al. Microarray expression profiling and functional characterization of *AtTPS* genes: duplicated *Arabidopsis thaliana* sesquiterpene synthase genes *At4g13280* and *At4g13300* encode root-specific and wound-inducible (*Z*)-γ-bisabolene synthases. *Arch Biochem Biophys*. 2006;**448**:104-16.

13. Vaughan MM, Wang Q, Webster FX. et al. Formation of the unusual semivolatile diterpene rhizathalene by the *Arabidopsis* class I terpene synthase TPS08 in the root stele is involved in defense against belowground herbivory. *Plant Cell*. 2013;**25**:1108-25.

14. Chen F, Tholl D, D'Auria JC. et al. Biosynthesis and emission of terpenoid volatiles from Arabidopsis flowers. *Plant Cell*. 2003;**15**:481-94.

15. Tholl D, Chen F, Petri J. et al. Two sesquiterpene synthases are responsible for the complex mixture of sesquiterpenes emitted from Arabidopsis flowers. *Plant J*. 2005;**42**:757-71.

16. Martin DM, Aubourg S, Schouwey MB. et al. Functional annotation, genome organization and phylogeny of the grapevine (*Vitis vinifera*) terpene synthase gene family based on genome assembly, FLcDNA cloning, and enzyme assays. *BMC Plant Biol*. 2010;**10**:226.

17. Külheim C, Padovan A, Hefer C. et al. The *Eucalyptus* terpene synthase gene family. *BMC Genomics*. 2015;**16**:450.

18. Liu G, Yang M, Yang X. et al. Five TPSs are responsible for volatile terpenoid biosynthesis in *Albizia julibrissin*. *J Plant Physiol*. 2021;**258-259**:153358.

19. Jiang Y, Qian R, Zhang W. et al. Composition and biosynthesis of scent compounds from sterile flowers of an ornamental plant *Clematis florida* cv. ‘Kaiser’. *Molecules*. 2020;**25**:1711.

20. Gao F, Liu B, Li M. et al. Identification and characterization of terpene synthase genes accounting for volatile terpene emissions in flowers of *Freesia* x *hybrida*. *J Exp Bot*. 2018;**69**:4249-65.

21. Bao T, Kimani S, Li Y. et al. Allelic variation of terpene synthases drives terpene diversity in the wild species of the *Freesia* genus. *Plant Physiol*. 2023;**192**:2419-35.

22. Booth JK, Page JE, Bohlmann J. Terpene synthases from *Cannabis sativa*. *PLoS One*. 2017;**12**:e0173911.

23. Zager JJ, Lange I, Srividya N. et al. Gene networks underlying cannabinoid and terpenoid accumulation in Cannabis. *Plant Physiol*. 2019;**180**:1877-97.

24. Booth JK, Yuen MMS, Jancsik S. et al. Terpene synthases and terpene variation in *Cannabis sativa*. *Plant Physiol*. 2020;**184**:130-47.

25. He J, Verstappen F, Jiao A. et al. Terpene synthases in cucumber (*Cucumis sativus*) and their contribution to herbivore-induced volatile terpenoid emission. *New Phytol*. 2022;**233**:862-77.

26. Yang P, Zhao HY, Wei JS. et al. Chromosome‐level genome assembly and functional characterization of terpene synthases provide insights into the volatile terpenoid biosynthesis of *Wurfbainia villosa*. *Plant J*. 2022;**112**:630-45.

27. Zhan C, Lei L, Guo H. et al. Disease resistance conferred by components of essential chrysanthemum oil and the epigenetic regulation of *OsTPS1*. *Sci China Life Sci*. 2023;**66**:1108-18.

28. Yuan JS, Köllner TG, Wiggins G. et al. Molecular and genomic basis of volatile‐mediated indirect defense against insects in rice. *Plant J*. 2008;**55**:491-503.

29. Chen H, Kollner TG, Li G. et al. Combinatorial evolution of a terpene synthase gene cluster explains terpene variations in *Oryza*. *Plant Physiol*. 2020;**182**:480-92.

30. Kamolsukyeunyong W, Sukhaket W, Pitija K. et al. Rice sesquiterpene plays important roles in antixenosis against brown planthopper in rice. *Plants (Basel)*. 2021;**10**:1049.

31. Cheng AX, Xiang CY, Li JX. et al. The rice (*E*)-β-caryophyllene synthase (OsTPS3) accounts for the major inducible volatile sesquiterpenes. *Phytochemistry*. 2007;**68**:1632-41.

32. Kiryu M, Hamanaka M, Yoshitomi K. et al. Rice terpene synthase 18 (*OsTPS18*) encodes a sesquiterpene synthase that produces an antibacterial (*E*)-nerolidol against a bacterial pathogen of rice. *J Gen Plant Pathol*. 2018;**84**:221-9.

33. Chen X, Chen H, Yuan JS. et al. The rice terpene synthase gene *OsTPS19* functions as an (*S*)‐limonene synthase *in planta*, and its overexpression leads to enhanced resistance to the blast fungus *Magnaporthe oryzae*. *Plant Biotechnol J*. 2018;**16**:1778-87.

34. Kiyama H, Matsunaga A, Suzuki G. et al. Monoterpene geraniol produced by rice terpene synthase 21 suppresses the expression of cell-division related genes in the rice bacterial pathogen, *Xanthomonas oryzae* pv. *oryzae*. *Physiol Mol Plant Pathol*. 2021;**115**:101673.

35. Yoshitomi K, Taniguchi S, Tanaka K. et al. Rice terpene synthase 24 (*OsTPS24*) encodes a jasmonate-responsive monoterpene synthase that produces an antibacterial γ-terpinene against rice pathogen. *J Plant Physiol*. 2016;**191**:120-6.

36. van Schie CCN, Haring MA, Schuurink RC. Tomato linalool synthase is induced in trichomes by jasmonic acid. *Plant Mol Biol*. 2007;**64**:251-63.

37. Bohlmann J, Martin D, Oldham NJ. et al. Terpenoid secondary metabolism in *Arabidopsis thaliana*: cDNA cloning, characterization, and functional expression of a myrcene/(*E*)-β-ocimene synthase. *Arch Biochem Biophys*. 2000;**375**:261-9.

38. Chen F, Ro DK, Petri J. et al. Characterization of a root-specific Arabidopsis terpene synthase responsible for the formation of the volatile monoterpene 1,8-cineole. *Plant Physiol*. 2004;**135**:1956-66.

39. Roos J, Bejai S, Mozuraitis R. et al. Susceptibility to *Verticillium longisporum* is linked to monoterpene production by TPS23/27 in Arabidopsis. *Plant J*. 2015;**81**:572-85.

40. Huang M, Abel C, Sohrabi R. et al. Variation of herbivore-induced volatile terpenes among Arabidopsis ecotypes depends on allelic differences and subcellular targeting of two terpene synthases, TPS02 and TPS03. *Plant Physiol*. 2010;**153**:1293-310.

41. Fäldt J, Arimura GI, Gershenzon J. et al. Functional identification of *AtTPS03* as (*E*)-β-ocimene synthase: a monoterpene synthase catalyzing jasmonate- and wound-induced volatile formation in *Arabidopsis thaliana*. *Planta*. 2003;**216**:745-51.

42. Abbas F, Ke Y, Zhou Y. et al. Molecular cloning, characterization and expression analysis of *LoTPS2* and *LoTPS4* involved in floral scent formation in oriental hybrid *Lilium* variety ‘Siberia’. *Phytochemistry*. 2020;**173**:112294.

43. Abbas F, Ke Y, Yu R. et al. Functional characterization and expression analysis of two terpene synthases involved in floral scent formation in *Lilium* ‘Siberia’. *Planta*. 2019;**249**:71-93.

44. Huang H, Kuo YW, Chuang YC. et al. *Terpene synthase-b* and *terpene synthase-e/f* genes produce monoterpenes for *Phalaenopsis bellina* floral scent. *Front Plant Sci*. 2021;**12**:700958.

45. Günnewich N, Page JE, Köllner TG. et al. Functional expression and characterization of trichome-specific (-)-limonene synthase and (+)-α-pinene synthase from *Cannabis sativa*. *Nat Prod Commun*. 2007;**2**:223-32.

46. Livingston SJ, Quilichini TD, Booth JK. et al. Cannabis glandular trichomes alter morphology and metabolite content during flower maturation. *Plant J*. 2020;**101**:37-56.

47. Qiao D, Tang M, Jin L. et al. A monoterpene synthase gene cluster of tea plant (*Camellia sinensis*) potentially involved in constitutive and herbivore-induced terpene formation. *Plant Physiol Biochem*. 2022;**184**:1-13.

48. Wang X, Gao Y, Wu X. et al. High-quality evergreen azalea genome reveals tandem duplication-facilitated low-altitude adaptability and floral scent evolution. *Plant Biotechnol J*. 2021;**19**:2544-60.

49. Yang S, Wang N, Kimani S. et al. Characterization of terpene synthase variation in flowers of wild *Aquilegia* species from northeastern Asia. *Hortic Res*. 2022;**9**:uhab020.

50. Lu G, Zhang T, He Y. et al. Virus altered rice attractiveness to planthoppers is mediated by volatiles and related to virus titre and expression of defence and volatile-biosynthesis genes. *Sci Rep*. 2016;**6**:38581.

51. Falara V, Alba JM, Kant MR. et al. Geranyllinalool synthases in Solanaceae and other angiosperms constitute an ancient branch of diterpene synthases involved in the synthesis of defensive compounds. *Plant Physiol*. 2014;**166**:428-41.

52. Matsuba Y, Nguyen TTH, Wiegert K. et al. Evolution of a complex locus for terpene biosynthesis in *Solanum*. *Plant Cell*. 2013;**25**:2022-36.

53. Schilmiller AL, Schauvinhold I, Larson M. et al. Monoterpenes in the glandular trichomes of tomato are synthesized from a neryl diphosphate precursor rather than geranyl diphosphate. *Proc Natl Acad Sci U S A*. 2009;**106**:10865-70.

54. Herde M, Gärtner K, Köllner TG. et al. Identification and regulation of TPS04/GES, an *Arabidopsis* geranyllinalool synthase catalyzing the first step in the formation of the insect-induced volatile C16-homoterpene TMTT. *Plant Cell*. 2008;**20**:1152-68.

55. Yamaguchi S, Sun TP, Kawaide H. et al. The *GA2* locus of *Arabidopsis thaliana* encodes *ent*-kaurene synthase of gibberellin biosynthesis. *Plant Physiol*. 1998;**116**:1271-8.

56. Helliwell CA, Sullivan JA, Mould RM. et al. A plastid envelope location of *Arabidopsis* *ent*-kaurene oxidase links the plastid and endoplasmic reticulum steps of the gibberellin biosynthesis pathway. *Plant J*. 2001;**28**:201-8.

57. Xu M, Wilderman PR, Peters RJ. Following evolution's lead to a single residue switch for diterpene synthase product outcome. *Proc Natl Acad Sci U S A*. 2007;**104**:7397-401.

58. Tezuka D, Ito A, Mitsuhashi W. et al. The rice *ent*-KAURENE SYNTHASE LIKE 2 encodes a functional *ent*-beyerene synthase. *Biochem Biophys Res Commun*. 2015;**460**:766-71.

59. Wilderman PR, Xu M, Jin Y. et al. Identification of *syn*-pimara-7,15-diene synthase reveals functional clustering of terpene synthases involved in rice phytoalexin/allelochemical biosynthesis. *Plant Physiol*. 2004;**135**:2098-105.

60. Otomo K, Kanno Y, Motegi A. et al. Diterpene cyclases responsible for the biosynthesis of phytoalexins, momilactones A, B, and oryzalexins A-F in rice. *Biosci Biotechnol Biochem*. 2004;**68**:2001-6.

61. Kanno Y, Otomo K, Kenmoku H. et al. Characterization of a rice gene family encoding type-A diterpene cyclases. *Biosci Biotechnol Biochem*. 2006;**70**:1702-10.

62. Cho EM, Okada A, Kenmoku H. et al. Molecular cloning and characterization of a cDNA encoding *ent*‐cassa‐12,15‐diene synthase, a putative diterpenoid phytoalexin biosynthetic enzyme, from suspension‐cultured rice cells treated with a chitin elicitor. *Plant J*. 2004;**37**:1-8.

63. Nemoto T, Cho EM, Okada A. et al. Stemar‐13‐ene synthase, a diterpene cyclase involved in the biosynthesis of the phytoalexin oryzalexin S in rice. *FEBS Lett*. 2004;**571**:182-6.

64. Morrone D, Hillwig ML, Mead ME. et al. Evident and latent plasticity across the rice diterpene synthase family with potential implications for the evolution of diterpenoid metabolism in the cereals. *Biochem J*. 2011;**435**:589-95.

65. Morrone D, Jin Y, Xu M. et al. An unexpected diterpene cyclase from rice: functional identification of a stemodene synthase. *Arch Biochem Biophys*. 2006;**448**:133-40.

66. RJ B, JAD Z. Comparison of *ent*-kaurene synthetase A-activity and B-activity in cell-free-extracts from young tomato fruits of wild type and *gib*-*1*, *gib*-*2*, and *gib*-*3* tomato plants. *J Plant Growth Regul*. 1990;**9**:237-42.

67. Otomo K, Kenmoku H, Oikawa H. et al. Biological functions of *ent*- and *syn*-copalyl diphosphate synthases in rice: key enzymes for the branch point of gibberellin and phytoalexin biosynthesis. *Plant J*. 2004;**39**:886-93.

68. Prisic S, Xu M, Wilderman PR. et al. Rice contains two disparate *ent*-copalyl diphosphate synthases with distinct metabolic functions. *Plant Physiol*. 2004;**136**:4228-36.
